# Assessing the Covalent Attachment and Energy Transfer Capabilities of Upconverting Phosphors With Cofactor Containing Bioactive Enzymes

**DOI:** 10.3389/fchem.2020.613334

**Published:** 2020-12-21

**Authors:** Letitia Burgess, Hannah Wilson, Alex R. Jones, Sam Hay, Louise S. Natrajan

**Affiliations:** ^1^Department of Chemistry, School of Natural Sciences, The University of Manchester, Manchester, United Kingdom; ^2^Manchester Institute of Biotechnology, The University of Manchester, Manchester, United Kingdom; ^3^Photon Science Institute, The University of Manchester, Manchester, United Kingdom

**Keywords:** lanthanides, upconversion, luminescence, protein conjugation, energy transfer, enzyme kinetics

## Abstract

Upconverting phosphors (UCPs) convert multiple low energy photons into higher energy emission via the process of photon upconversion and offer an attractive alternative to organic fluorophores for use as luminescent probes. Examples of biosensors utilizing the apparent energy transfer of UCPs and nanophosphors (UCNPs) with biomolecules have started to appear in the literature but very few exploit the covalent anchoring of the biomolecule to the surface of the UCP to improve the sensitivity of the systems. Here, we demonstrate a robust and versatile method for the covalent attachment of biomolecules to the surface of a variety of UCPs and UCNPs in which the UCPs were capped with functionalized silica in order to provide a surface to covalently conjugate biomolecules with surface-accessible cysteines. Variants of BM3Heme, cytochrome C, glucose oxidase, and glutathione reductase were then attached via maleimide-thiol coupling. BM3Heme, glucose oxidase, and glutathione reductase were shown to retain their activity when coupled to the UCPs potentially opening up opportunities for biosensing applications.

## Introduction

Lanthanide-doped upconverting phosphors (UCPs) have attracted considerable attention in recent years as an important and versatile class of luminescent nanoparticles (Zhou et al., 2015b). Upconversion (UC) is an anti-Stokes process involving the sequential absorption of multiple low-energy photons and subsequent emission of one higher-energy photon, facilitated by the ladder-like arrangement of long-lived energy levels found in lanthanide ions (Haase and Schäfer, 2011). This means that, in contrast to conventional fluorophores, upconverting materials can achieve visible emission with excitation in the near-Infra-Red (nIR) region of the electromagnetic spectrum.

While the upconversion phenomenon has recently been observed in discrete molecular complexes (Aboshyan-Sorgho et al., 2011; Blackburn et al., 2012; Suffren et al., 2013; Nonat et al., 2016; Charbonnière, 2018; Golesorkhi et al., 2019, 2020; Woodward et al., 2019; Nonat and Charbonnière, 2020), UC is most efficiently achieved by doping lanthanide ions into a low-phonon crystalline matrix (e.g., NaYF_4_, Gd_2_O_2_S), where non-radiative decay can be minimized (Haase and Schäfer, 2011). The past two decades have seen the successful translation of these materials from the bulk solid to nanocrystals with controlled size, morphology and surface-properties, enabling an increasingly diverse range of applications (Zhou et al., 2015a). In particular, the ability to modify the surface of UCPs in a stable aqueous dispersion lends itself well to biological applications including bio-imaging, sensing, and photodynamic therapy (Wang et al., 2005; Chatterjee et al., 2008; Idris et al., 2012).

In biological media, excitation in the nIR enables deeper penetration through tissue and an improved signal-to-noise via the avoidance of background auto-fluorescence (Haase and Schäfer, 2011). In addition to these merits, UCPs also offer negligible photobleaching, no photoblinking and low cyto-toxicity (Haase and Schäfer, 2011; Gnach et al., 2015). Furthermore, excitation at a single wavelength can produce simultaneous emission from a number of different doped lanthanide ions, with characteristic emission bands easily resolved by wavelength or by lifetime (typically μs-ms) (Wang and Liu, 2008; Eliseeva and Bünzli, 2010).

Many UCP biosensing systems developed in the literature to date utilize luminescence resonance energy transfer (LRET). This is a distance-dependent energy transfer process that only occurs when a donor and acceptor moiety are within the Förster radius (<10 nm) of each other (Lakowicz, 2006). For example, UCP donor emission can be quenched by an LRET acceptor that is brought into range by the presence of the target analyte, as with labeled antibodies in a sandwich immunoassay (Wang et al., 2009). Alternatively, UCP donor emission can be restored by separation from an LRET acceptor by analyte-induced cleavage of a connection between the two (Wang et al., 2012).

Rather than modulating the separation distance of donor and acceptor, we have recently reported the development of systems that exploit a change in the spectral overlap of a biomolecule acceptor with the UCP. This approach has often been applied to the detection of metal ions, whereby the absorption maximum of a moiety on the UCP surface is modulated by complexation of a metal ion (Liu et al., 2011; Peng et al., 2015). Interestingly, there are a number of active biomolecules with chromophoric co-factors that could be monitored in a similar way (Burgess et al., 2020).

Our initial work showed that UCPs could be used to monitor enzyme turnover for pentaerythritol tetranitrate reductase (PETNR), a flavin-dependent enzyme with spectrally distinct redox states, and we later expanded the scope to the detection of a number of PETNR substrate analytes (Harvey et al., 2014; Oakland et al., 2017). In these earlier studies where the enzyme was added to the UCP containing solution, we were unable to conclusively assign the mechanism of apparent energy transfer (AET) between the UCP donor and PETNR-flavin mononucleotide (FMN) acceptor. However, in order to maximize the energy transfer efficiency in such systems, it is advantageous for the biomolecule acceptor to be attached to the UCP surface, thereby placing the donor and acceptor within (or close to) the Förster radius. Our more recent work has therefore reported the covalent attachment of PETNR to UCPs, showing that catalytic activity is retained upon attachment, and that it is still possible to ratiometrically monitor the enzyme redox state and reversibly detect substrates (Natrajan et al., 2020).

In considering the covalent attachment of active biomolecules to UCPs, it is important to acknowledge the dependence of activity on a highly specific structure, such as the shape of an enzyme active site, or the position of an active cofactor (Sapsford et al., 2013). Bioconjugation must therefore ensure minimal structural disruption and take into account the resulting orientation of the bound enzyme or biomolecule on the UCP surface.

Methods that have been used to attach active enzymes have appeared in the literature, such as using a DNA linker to attach horseradish peroxidase (Lu et al., 2015). Parallels can also be drawn with the attachment of non-catalytic proteins, such as the use of carbodiimide chemistry to attach a folate binding protein (Arppe et al., 2015), and a number of different antibodies (Sedlmeier and Gorris, 2015). However, although commonly utilized bioconjugation strategies should be feasible with UCPs, studies in this area are lacking and there remains no reliable bio-toolbox for the attachment of active enzymes to UCPs, particularly when looking to optimize separation distances for efficient LRET.

Here we expand on our reports of covalently bound active PETNR by screening a number of conjugation methods to contribute to the aforementioned bio-toolbox. Starting with organic dye models, we establish the importance of using several characterization methods in analyzing the success of a reaction. We then use a green fluorescent protein (GFP) to screen bioconjugation methods, before selecting one robust and versatile method for the attachment of a variety of active biomolecules. Finally, we prove that the activity of these proteins is retained upon conjugation, thus presenting an opportunity for the development of highly sensitive UCP-biomolecule sensors.

## Materials and Methods

### Materials

Hexagonal phase Gd2O2S:Yb,Tm (PTIR475) and Gd2O2S:Yb,Er (PTIR545) upconverting phosphors were donated by Phosphor Technology Ltd. See Supplementary Figure 30 for powder X-ray diffraction spectra. Enhanced GFP was prepared as described previously (Natrajan et al., 2020). The heme domain of cytochrome P450 BM3/CYP102A1 and *N*-palmitoylglycine (NPG) were prepared as described previously (Brenner et al., 2007) and donated by the Munro group (The University of Manchester). Glucose oxidase and glutathione reductase were purchased from Sigma-Aldrich. Sulfo-SMCC was purchased from Thermo-Fischer Scientific. 6-maleimidohexanoic acid (MHA) was purchased from Alfa Aesar. SAMSA fluorescein (SF) was purchased from Life Technologies Limited. Fluorescein maleimide was purchased from Vector Laboratories Limited. Carboxyethylsilanetriol di-sodium salt was purchased from Fluorochem Limited. All other solvents, reagents and biomolecules were purchased from Sigma-Aldrich. All reagents and solvents were used as received. Deionised (DI) water was obtained from a Millipore Synergy water purification system.

### Characterization Methods

Emission spectra were recorded in 1 cm^3^ quartz cuvettes using an Edinburgh Instrument FP920 phosphorescence lifetime spectrometer (with single 300 mm focal length excitation and emission monochromators in Czerny Turner configuration) equipped with a 45 mW continuous wave (CW) 980 nm diode laser (Edinburgh Instruments) and a red sensitive photomultiplier in peltier (air cooled) housing (Hamamatsu R928P). No detector correction files were used when collecting the emission spectra in order to observe the 475 nm blue emission band and the nIR emission band at 800 nm of the upconverting phosphors on the same intensity scale and to observe the quenching of the 475 nm emission band during the experiments (i.e., without having to expand the blue region of the spectra manually).

TEM images were obtained on a 200 kV Phillips Microscope on carbon coated copper grids and were analyzed using the Gatan3 Digital Micrograph Software Package. The TEM grids were purchased from Agar Scientific Limited and were prepared by the drop cast method using a dilute sample of the UCPs and air-dried.

Fourier transform Infrared (FTIR) spectra were obtained using solid samples on a Bruker alpha FT-IR spectrometer and the data was analyzed using the OMNIC software.

Raman Spectra were recorded on a Horiba Xplora plus confocal Raman spectrometer on glass microscope slides, data were analyzed using the LabSpec software.

The dynamic light scattering (DLS) size and zeta potential analysis was performed on a 1 mg/mL aqueous solution of the UCPs. Both measurements were recorded in disposable folded capillary cells (DST1070) using a 633 nm laser as the excitation source on a Malvern Zetasizer nano ZS instrument. The data were analyzed using the Zetasizer programme and the results quoted are the average of three measurements.

UV visible spectra were recorded on a 1 mg/mL aqueous solution of UCPs on a Cary 60 Spectrophotometer (Aglient) using a 1 cm^3^ quartz cuvette, the data were analyzed using the Cary WinUV software.

Enzyme kinetic studies were carried out in a Belle glovebox under an N_2(g)_ atmosphere, using a Cary 60 bio UV-Vis spectrometer and the Cary kinetics program.

Reflectance spectra were recorded using a Perkin Elmer Lambda 1050 UV/Vis/nIR spectrometer with a tungsten lamp and a 150 mm InGaAs integrating sphere. Data were analyzed using the Perkin Elmer UV WinLab programme.

The microanalytical department at The University of Manchester carried out thermogravimetric analysis (TGA) from 35 to 600°C on dry UCP samples in air using a Mettler Toledo TGA/DSC1 Star System.

### Synthetic Procedures

#### General Procedure for the Synthesis of Silica Capped Industry Upconverting Phosphors

Silica capped **(silica475 and silica545);** (3-aminopropyl)triethoxysilane capped **(APTES475 and APTES545);** (3-mercaptopropyl)trimethoxysilane capped **(MPTMS475 and MPTMS545)**.

Industry procured gadolinium oxysulfide of hexagonal (β*-*) phase Gd_2_O_2_S (Supplementary Figure 30) upconverting phosphors (PTIR-UCPs) (15 mg) were dispersed in a mixture of cyclohexane (40 mL) and Igepal CO-520 (1.5 mL, 3.39 mmol) before 25% ammonium hydroxide (0.5 mL) was added. After stirring for 60 min TEOS (60 μL, 0.00039 mmol) was added. After a further 60 min, APTES (60 μL, 0.00029 mmol) or MPTMS (60 μL, 0.00031 mmol) was optionally added to give further functionalisation as appropriate. The reaction mixture was then stirred for 48 h in a sealed flask at room temperature. Acetone (15 mL) was added to cause the nanoparticles to precipitate. The precipitate was collected by centrifugation (60 min, 4,000 rpm). The pellet was washed with acetone (2 × 25 mL) and then with ethanol:water (2:1, 30 mL) and collected by centrifugation (60 min, 4,000 rpm).

Triethoxysilane-PEG-maleimide capped **(MPS475)**

As prepared above, silica475 (20 mg) were dispersed in deionized water:ethanol (22 mL, 1:10 v/v) before 25% ammonium hydroxide (1 mL) was added. Triethoxysilane-PEG-maleimide (20 mg, 0.004 mmol) was then dissolved in deionized water:ethanol (1.5 mL, 2:1 v/v). The two solutions were combined and the reaction mixture was stirred overnight in a sealed flask at room temperature. The UCPs were collected by centrifugation and washed with ethanol (2 × 20 mL) then water (1 × 20 mL).

#### General Procedure for the Solvo(hydro)Thermal Synthesis of Functionalized NaYF_4_:Yb,Tm Upconverting Nanoparticles

According to modifications of our previous works (Oakland et al., 2017), an aqueous solution of NaOH was combined with solutions of organic ligands in either ethanol or water. Oleic acid was then added (if required), followed by 10 min of stirring to form a homogenous solution. Two further additions were each followed by 10 min of stirring: (a) an aqueous solution of lanthanide salts; (b) an aqueous solution of NaF (dropwise). The resulting mixture was transferred to a Teflon lined reaction vessel and heated at 120°C for 30 min, before increasing the temperature to 200°C for 5 h. After cooling, the solution was centrifuged to obtain the product, and washed with ethanol three times.

Exact reagent quantities are given below:

**6-aminohexanoic acid, 6-maleimidohexanoic acid and oleic acid capped (AHAMHAOAYbTm):** 5 mL aqueous solution of NaOH (1 g, 24.4 mmol) and AHA (5 g, 38 mmol); 8.5 mL ethanol solution of MHA (2 g, 9.5 mmol); 17 mL of OA; 1.2 mL aqueous solution of LnCl_3_.6H_2_O (0.5 M, 20 % Yb, 0.2 % Tm, 79.8 % Y); 4 mL aqueous solution of NaF (168 mg, 4 mmol).

**6-maleimidohexanoic acid capped (MHAYbTm):** 4 mL aqueous solution of NaOH (1 g, 24.4 mmol); 25 mL ethanol solution of MHA (11.4 g, 54 mmol); 1.2 mL aqueous solution of LnCl_3_.6H_2_0 (0.5 M, 20 % Yb, 0.2 % Tm, 79.8 % Y); 4 mL aqueous solution of NaF (168 mg, 4 mmol).

**Dimercaptosuccinic acid capped (DMSAYbTm):** 2 mL aqueous solution of NaOH (0.2 g, 5 mmol); 5 mL ethanol solution of DMSA (0.7697 g, 4.22 mmol); 0.24 mL aqueous solution of LnCl_3_.6H_2_0 (0.5 M, 20 % Yb, 0.2 % Tm, 79.8 % Y); 1 mL aqueous solution of NaF (33.58 mg, 0.8 mmol).

**Cysteine capped (cysteineYbTm):** 3 mL aqueous solution of NaOH (0.940 g, 7.76 mmol); 18.5 mL ethanol solution of cysteine (4.9273 g, 40.5 mmol); 1.2 mL aqueous solution of LnCl_3_.6H_2_0 (0.5 M, 20 % Yb, 0.2 % Tm, 79.8 % Y); 4 mL aqueous solution of NaF (168 mg, 4 mmol).

**Polyethylenimine capped (PEIYbTm):** Y(NO_3_)_3_.6H_2_O (0.0715 g, 0.1868 mmol), Yb(NO_3_)_3_.6H_2_O (0.0281 g, 0.0625 mmol) and Tm(NO_3_)_3_.6H_2_O (0.0033 g, 0.0075 mmol) were dissolved in deionised water (9 mL) before PEI (340 mg, 0.0034 mmol) was added and the reaction mixture stirred for 5 min. Then an aqueous solution (9 mL) of NaF (126 mg, 3 mmol) was added with vigorous stirring. Ethanol (9 mL) was added and the reaction mixture stirred for 10 min before a second addition of ethanol (9 mL) was added and the contents were transferred to a Teflon lined reaction vessel and heated at 120°C for 30 min before increasing the temperature to 200°C for 17 h. The solution was centrifuged after cooling. Washed with ethanol (3 × 10 mL).

#### General Procedures for the Covalent Attachment of GFP to the Surface of Functionalized UCPs

An aqueous solution of functionalized UCPs (1 mL) was diluted with PBS buffer (1 mL, 100 mM, pH 7.4), before the addition of GFP. Mixtures were left under gentle agitation at 4°C for 24 h. Products were collected by centrifugation (10 min, 10,000 rpm) and washed three times with PBS. The mass of UCPs in the initial 1 mL dispersion is given in brackets below for all conjugations.

The following were all reacted with 0.004 μmol of GFP (300 μL of 13 μM stock solution): **AHAMHAOAYbTm_GFP** (12.1 mg); **MHAYbTm_GFP** (18.6 mg); **DMSAYbTm_GFP** (8.3 mg); **CysteineYbTm_GFP** (17.7 mg).

The following were both reacted with 0.01 μmol of GFP (500 μL of 33 μM stock solution): **MPS475_GFP** (1.8 mg); **MPTMS475_GFP** (10.6 mg).

The following were reacted with 0.02 μmol of GFP (400 μL of 40 μM stock solution), which had previously been combined at room temperature for 30 min with 100 μL of sulfo-SMCC cross-linking reagent (1 mg, 2.3 μmol): **APTES475_GFP** (52.1 mg); **PEIYbTm** (39.3 mg).

### General Procedures for the Covalent Attachment of Other Biomolecules to the Surface of Functionalized UCPs

Sulfo-SMCC (2 mg, 4.6 μmol) was dissolved in deionized water (200 μL) then diluted with PBS buffer (1 mL, pH 7.4, 100 mM). After addition of the biomolecule (see quantities below), the mixture was left at room temperature for 30 min. An aqueous solution (1 mL) of APTES475 or APTES545 (10 mg) was added, and the mixture left under gentle agitation at 4°C for 24 h. Products were collected by centrifugation (10 min, 10,000 rpm) and washed three times with PBS.

Quantities of biomolecule added were as follows:

0.04 μmol **BM3Heme** (500 μL of 75 μM solution); 0.4 μmol **CytC** (5 mg); 0.014 μmol **glucose oxidase** (2.3 mg); 0.006 μmol **glutathione reductase** (500 μL of 1.5 mg/mL suspension).

## Results and Discussion

### Preliminary Screening With Organic Dyes

Given the large, variable size and complexity of biomolecules, covalent attachment methods were first investigated using a range of structurally more simple organic dyes that absorb visible light (structures shown in Supplementary Figure 1). Five dyes were selected based on their spectral overlap with UC emission (Supplementary Figure 2) and on the presence of functional groups ideal for different conjugation methods. We have previously demonstrated the ability of these organic dyes to act as apparent energy transfer (AET) acceptors in non-covalent systems with UCPs (Burgess et al., 2020).

Reactions are summarized in Table 1, with further experimental details provided in the Supplementary Information. All reactions used Gd_2_SO_4_ upconverting phosphors, referred to by the wavelength of their key upconversion emission band as PTIR475 and PTIR545 for Tm^3+^ and Er^3+^ doped crystals, respectively. Functional groups were introduced via silica capping as indicated in Table 1.

**Table 1:** Summary of initial methods screened for the attachment of organic dyes to the surface of functionalized PTIR475 or PTIR545 upconverting phosphors.

**Dye**	**Further dye functionalisation**	**Functionalized silane capping on PTIR475 or PTIR545**	**Coupling Method**
Fluorescein isothiocyanate (FITC)	n/a	3-aminopropyl triethoxysilane (APTES)	Isothiocyanate + amine
Rhodamine B isothiocyanate (RBITC)			
Methyl red (MR)	Carbodiimide activation of carboxylic acid		Carbodiimide coupling
SAMSA fluorescein (SF)	Basic deprotection of thiol (acetyl group)		Thiol + maleimide (with a sulfo-SMCC crosslinker)
Fluorescein maleimide (FM)	n/a	3-mercaptopropyl trimethoxysilane (MPTMS)	Thiol + maleimide

**Figure 1:**
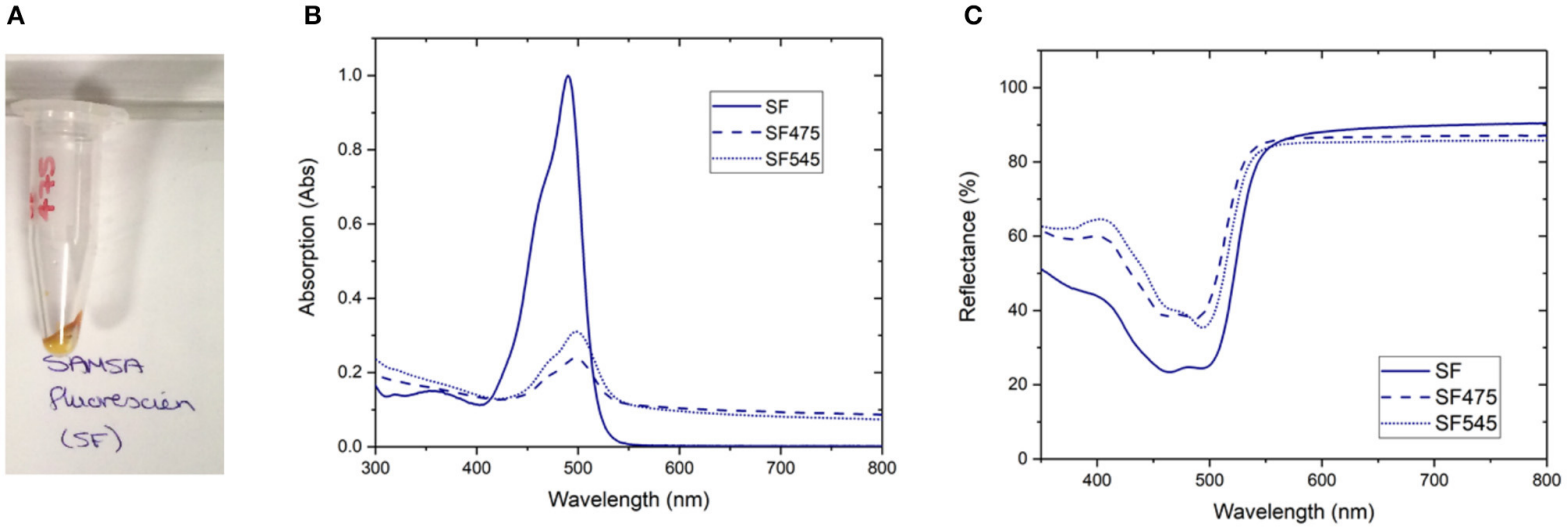
A selection of characterization data for the attachment of SAMSA fluorescein (SF) to PTIR475 or PTIR545 **(A)** photograph of the SF475 product, **(B)** solution UV-vis spectra for SF, SF475, and SF545, **(C)** reflectance spectra for SF, SF475, and SF545.

**Figure 2:**
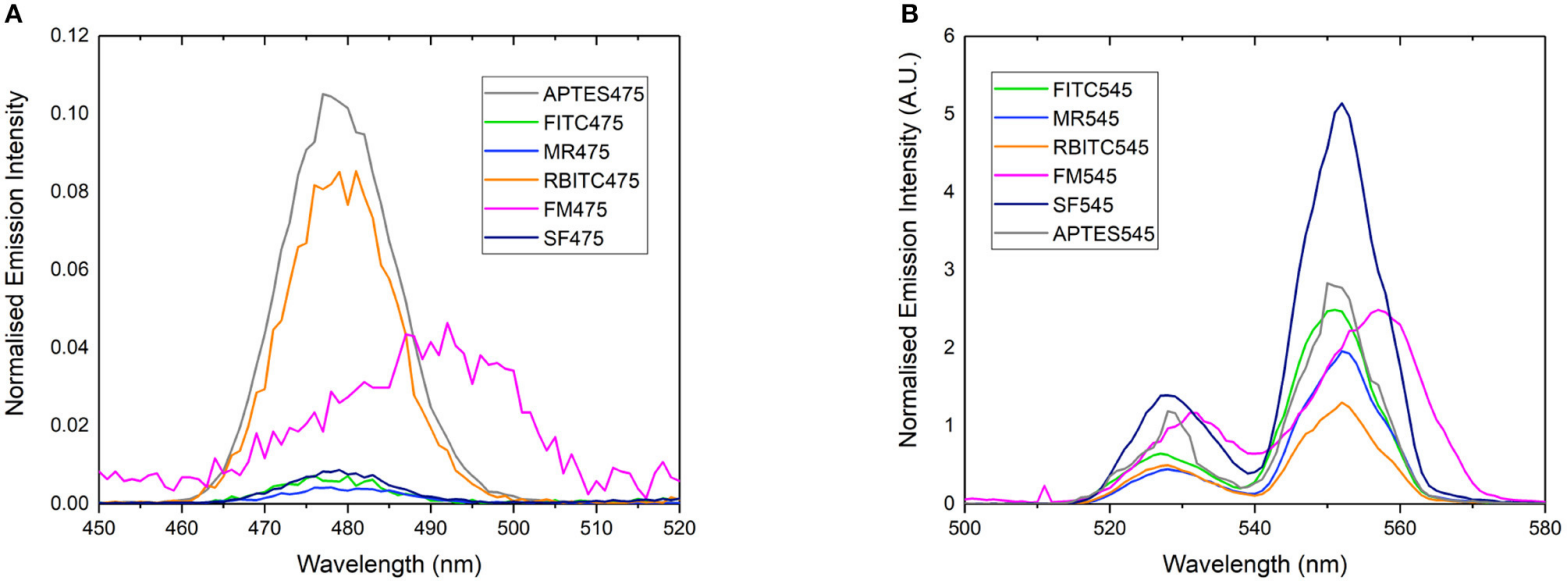
Normalized emission spectrum of **(A)** APTES475, FITC475, RBITC475, MR475, SF475, and FM475 showing only the 475 nm band and **(B)** APTES545, FITC545, RBITC545, MR545, SF545, and FM545 showing only the 545 nm band. All solutions were 1 mg/mL in 100 mM PBS pH 7.4. Excitation at 980 nm. All spectra are reported uncorrected for the detector response. Spectra are normalized relative to the unquenched emission bands at 800 or 660 nm (not shown here, see Supplementary Figure 7 for expanded spectra).

The four conjugation methods were all found to be successful and, most importantly, this screening process enabled us to identify the most appropriate characterization methods for determining this success. Full characterization was conducted using UV-vis solution spectroscopy, reflectance spectroscopy, fluorescence spectroscopy, IR spectroscopy, Raman spectroscopy, thermogravimetric analysis (TGA), dynamic light scattering (DLS), transmission electron microscopy (TEM), and visual inspection. A comprehensive summary of all data can be found in Supplementary Figures 4–12, with highlights illustrated in Figures 1, 2.

Visual inspection gave the first indication of successful attachment; the white UCP precursors became highly colored after the reaction workup (as seen for SF475 in Figure 1A, and other dyes in Supplementary Figure 4). Note that pellets were washed repeatedly until the supernatant was shown to contain no free dye by UV-vis absorption spectroscopy (Supplementary Figure 3), so the color is most likely to originate from organic dye covalently attached to the UCP surface.

Solution UV-vis spectra of the re-dispersed SF475 and SF545 products, as an example, clearly show absorption maxima corresponding to the dye molecule (Figure 1B). However, the absorption maximum is slightly shifted to lower energy compared to the starting material SF dye, which can be rationalized in terms of the coupling reaction used. Given the thiol ether was first deprotected then coupled with sulfo-SMCC (sulfosuccinimidyl 4-(*N*-maleimidomethyl)cyclohexane-1-carboxylate) in a thiol-malemide coupling, slight changes in the electronic structure of the aromatic system and solvatochromism in solution would account for the observed red shift. In addition, the UV-vis solution spectra also featured scattering attributed to the UCPs (~1–2 μM in size). In most other reactions, this obscured the identification of any dye absorption bands (Supplementary Figure 5). Scattering effects in solution can be avoided by conducting reflectance spectroscopy, where samples are drop cast and dried onto glass slides. The resulting spectra are inversely proportional to absorbance, and characteristic reflectance bands were observed for both SF475 and SF545 (Figure 1C) and all other dye reactions (Supplementary Figure 6). We concluded that reflectance offered superior characterization to solution UV-vis spectroscopy for these UCP dispersions.

Further characterization should be possible using IR spectroscopy by identifying key absorption bands in the reaction products. However, although correct functional group vibrations were present in the UCP-dye conjugate spectra, the overall poorer resolution prevented full spectral assignments, particularly in the fingerprint region (Supplementary Figure 8). Raman spectroscopy also yielded little useful information regarding conjugation, primarily due to the free dye spectra being reasonably featureless and pronounced lattice vibrations from the UCPs (Supplementary Figure 9).

Rather than identifying the dye itself, we also looked to characterize the resulting energy transfer from the UCP to the surface-bound dyes. It is clear from emission spectra (Figure 2) that the UC band is quenched in almost all dye-UCP conjugates when compared to the precursor APTES475 or APTES545 UCPs. Of note, the spectra of FM475 and FM545 (Figure 2) are bathochromically shifted by ca. 12 nm with respect to the parent MPTMS475 and MPTMS545 UCPs (See Supplementary Figure 29) and those functionalized with all the other dye molecules in this study. The exact reason behind this is not immediately obvious, but one possible reason is that in the FM conjugated systems, additional crystal field effects imposed by the different surface functionalisation with FM is more pronounced (Eliseeva and Bünzli, 2010; Hatanaka and Satoshi, 2014). Additionally, the upconverted emission spectrum of SF545 shows an apparent increase in intensity compared to the APTES545 precursor. Given that the corresponding spectra in the SF475 system show the expected decrease in emission intensity, this observation suggests that the normalization (to the UCNP 660 emission band) is giving an artificially inflated signal due to a small degree of spectral overlap of the FM dye with both 550 and 660 nm emission bands in the UCP (Burgess et al., 2020) (Supplementary Figure 2).

The success of the dye-attachment to the UCPs was additionally examined by DLS, TEM and/or TGA. However, negligible differences between the starting materials and products were observed in the DLS and TEM data, whilst the presence of several different organic moieties hindered accurate assignment of weight losses by TGA. Because the addition of a layer of organic dye will only impart a small effect on the hydrodynamic ratios in solution (DLS) and not significantly add to the scattering observable by TEM, these observations are not surprising. Conducting these measurements are nevertheless important to show that there has been no change to the bulk morphology and dispersion of the UCPs following conjugation reactions (Supplementary Figures 10–12). In different systems, the attachment of larger or more charged molecules, it may be possible to identify the presence of an additional capping layer via DLS or TEM. It may also be possible in some cases to quantify the amount of dye attached using TGA.

From this preliminary work, we demonstrated that the success of covalent attachment to UCPs requires analysis using the combination of several spectroscopic techniques. Here, reflectance UV-vis absorption spectroscopy, emission spectroscopy alongside visual inspection were found to be most useful. We note, in the case of IR spectroscopy, however, that the data may not always be as conclusive as expected, highlighting the importance of a comprehensive approach to characterization.

From here we moved on to screening coupling methods with a range of suitable proteins.

### Screening With GFP

Although proteins do not naturally contain either isothiocyanate or maleimide groups for bioconjugation reactions using these functional groups, the natural amino acids present include thiol, amine and carboxylic acid groups, which are available moieties for a variety of bioconjugation reactions. However, the prevalence of carboxylic acid and amine groups makes it unfeasible to target a single residue, leading to issues with non-specific orientation of the conjugated protein on the UCP surface. More specific conjugation can be achieved by targeting the thiol side-chain of cysteine residues. While these are often engaged in disulfide bridging within the tertiary structure of a biomolecule, certain surface cysteine residues are readily accessible for functionalization and the proteins herein, were chosen so as to take advantage of this fact. In cases, where the protein has no available surface cysteine group, protein engineering can be utilized to introduce an accessible surface cysteine residue at a specific location that can be optimized for bioconjugation with retention of catalytic activity.

**Scheme 1:**
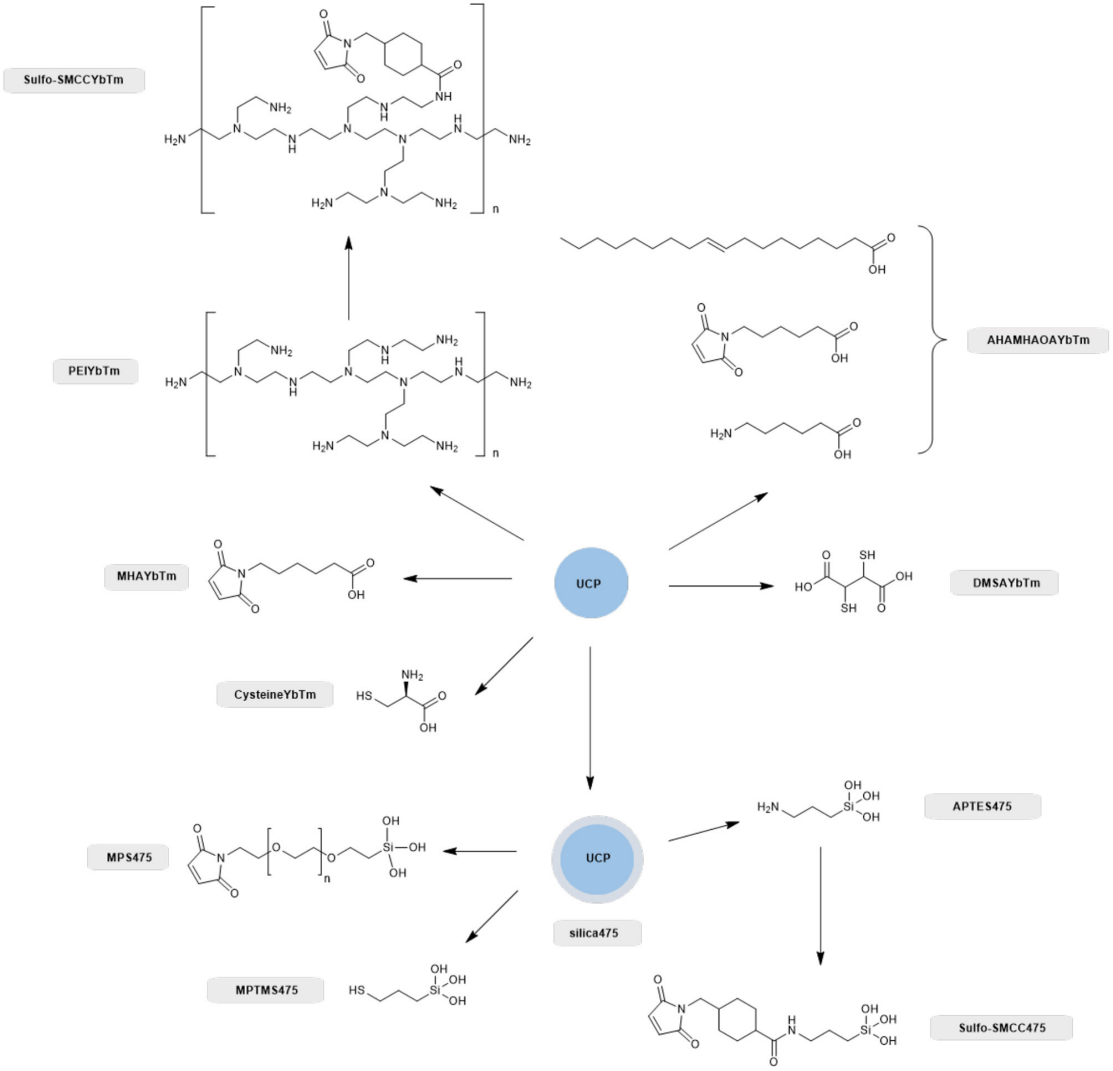
Overview of the different ligand systems investigated for the covalent attachment of GFP with compound names shown alongside. All systems that relied on a silica capping layer for the attachment of the thiol or maleimide functional group were derived from the PTIR UCPs (silica475), whereas synthesized nanoparticles directly capped with carboxylate containing thiol, amine and maleimide linkers negated the use of a silica capping layer and these were nanoparticles based on solvo(hydro)thermal syntheses as described previously (Oakland et al., 2017).

Therefore, we next screened the potential of a variety of UCPs in coupling to proteins with surface cysteine residues (Scheme 1). Enhanced GFP was chosen for the conjugation screening because it is a stable and well-characterized protein whose absorption profile overlaps with the 475 nm band of thulium doped UCPs (Burgess et al., 2020). In total, eight different UCPs and UCNPs were screened, as summarized in Scheme 1. Three UCPs possess surface thiol groups (DMSAYbTm, MPTMS475, and cysteineYbTm), three have surface maleimide groups (AHAMHAOAYbTm, MPS475, and MHAYbTm), and two have surface amine groups (PEIYbTm and APTES475). The surface amines were further functionalized with a sulfo-SMCC linker to convert the amine into a maleimide group. All reactions are classified as either thiol-thiol couplings, or thiol-maleimide couplings.

For all GFP conjugation reactions, the phosphor/nanophosphor was suspended in PBS (100 mM, pH 7.4) and left to react at 4°C under gentle agitation for 48 h. The reaction must be carried out at a pH between pH 6.5–pH 7.5 for it to be selective for the surface cysteine group (thiol containing side chain) over the lysine residues (amine containing side chain) in GFP. In this pH range, the thiol group of the cysteine is nucleophilic and will react with the maleimide group whereas the amine groups are protonated and unreactive. Outside of this pH range, the amines may be nucleophilic enough to compete with the thiol groups.

**Figure 3:**
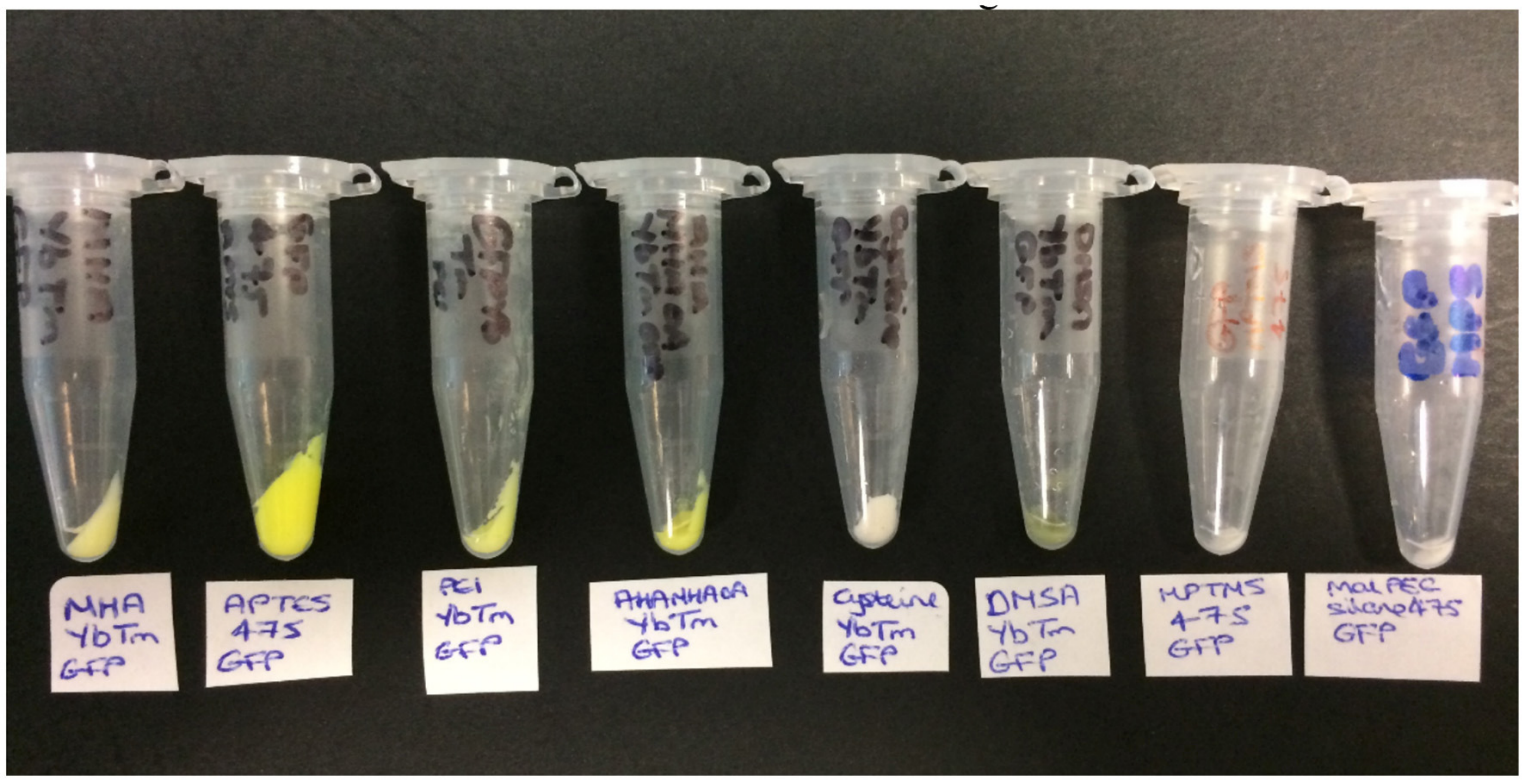
The products obtained from the covalent attachment of GFP reactions (from left to right): MHAYbTm_GFP, APTES475_GFP, PEIYbTm_GFP, AHAMHAOAYbTm_GFP, CysteineYbTm_GFP, DMSAYbTm_GFP, MPTMS475_GFP, and MPS475_GFP.

The product was then collected by centrifugation and washed with PBS until the supernatants were shown to be clear (free of unbound GFP) by solution UV-visible spectroscopy (Supplementary Figure 13). The color of the resulting pellet gave the first indication of whether the reaction has been a success, as shown in Figure 3. A green pellet is consistent with GFP being bound to the UCPs; therefore MHAYbTm, APTES475, PEIYbTm, AHAMHAOAYbTm, and DMSAYbTm are the most likely to have been covalently attached to GFP successfully. The pellets for cysteineYbTm, MPTMS475, and MPS475 remained white after work up, indicating attachment of either a very small amount of GFP, or none at all.

Based on these observations, maleimide capped phosphors are likely to be superior for bioconjugation. This is also seen in wider protein chemistry; commercial routes for labeling of biomolecules focuses on this maleimide-thiol chemistry and not thiol-thiol chemistry. This is due to the reversible formation of the thiol-thiol bond rendering this method less robust than desired.

The UCP-GFP conjugates were fully characterized using methods as with UCP-dye studies; a comprehensive summary is provided in the Supplementary Figures 13–21.

As observed with the UCP-dye conjugates, the quality of the data obtained for the UCP-GFP conjugates varied depending on the system in question, so that no single technique could be used to verify the success of the bioconjugation reaction. This point is highlighted in Table 2. Certain data show changes likely originating from successful GFP attachment (✓✓); some data show changes but cannot reliably be attributed to GFP (✓); some data show no changes at all (χ). Notably, the spectral data do not always align with the color changes observed by eye, which renders robust analysis of the relative success of UCP-GFP conjugation reactions challenging.

**Table 2:** An overview of the results obtained from the analytical analysis of all the UCP-GFP conjugates.

**UCP used *(avg. diameter of product by TEM)***	**Color**	**Emission**	**Solution UV-Vis**	**Reflectance**	**IR**	**Raman**	**DLS**	**TEM**	**TGA**
AHAMHAOAYbTm *(170 nm)*	✓✓	χ	✓✓	✓✓	✓	✓	✓	✓	✓
APTES475 *(0.8 μm)*	✓✓	✓✓	✓✓	✓✓	✓	✓	✓	✓✓	–
PEIYbTm *(80 nm)*	✓✓	χ	χ	✓✓	✓	✓	✓	✓	✓
DMSAYbTm *(640 nm)*	✓✓	✓	✓✓	χ	✓	✓	✓	✓	✓
MHAYbTm *(360 nm)*	✓✓	✓	χ	✓✓	✓	✓	✓	✓	χ
CysteineYbTm *(1.5 μm)*	χ	✓	χ	χ	χ	✓	✓	✓	✓
MPS475 *(1.2 μm)*	χ	χ	χ	χ	✓	χ	✓	✓	–
MPTMS475 *(1.0 μm)*	χ	✓	χ	χ	✓	χ	✓	✓	–

*A double tick indicates that there has been a change in the observable behavior of the system after the GFP attachment reaction and that it is likely to be due to GFP attachment, a single tick indicates that there has been a change in the observable behavior of the system after the GFP attachment reaction but that it cannot be conclusively determined to be due to GFP attachment, a cross indicates that there has been no change in the observable behavior of the system after the GFP attachment reaction and a dash means that this measurement could not collected for this system*.

Nevertheless, we believe that the characterization data for the reaction with APTES475 (highlighted row in Table 2) provides compelling evidence for a successful bioconjugation reaction. Our previous work with the covalent attachment of PETNR (Natrajan et al., 2020) utilized this same coupling method, and our results here are an encouraging indication of its suitability in comparison to other screened systems. From here, we moved on to studying the attachment of other active biomolecules (truncated heme domain of cytochrome P450 BM3/CYP102A1 (BM3Heme), cytochrome *c* (cytC), glucose oxidase (GO) and glutathione reductase (GR).

### Covalent Attachment of Active Proteins

**Figure 4:**
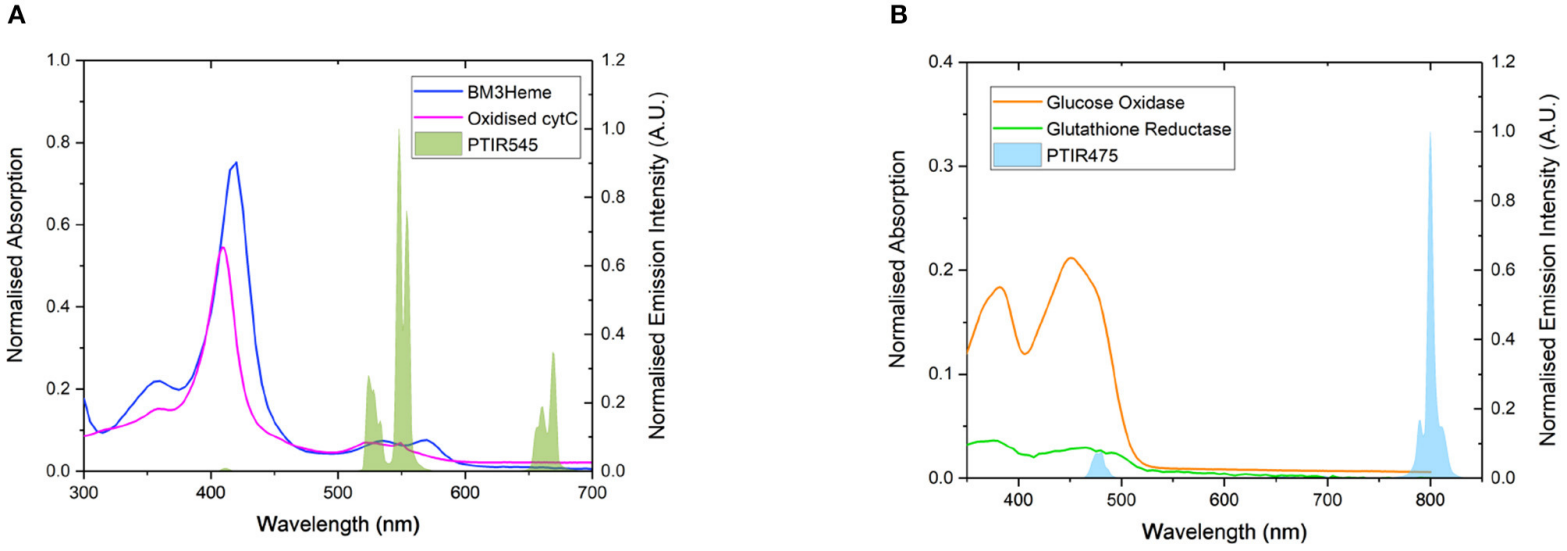
**(A)** The overlap of the absorption bands of BM3Heme (8 μM) in 100 mM PBS pH 7.4 and oxidized cytC (10 μM) in 100 mM TRIS pH 7 with the emission bands of PTIR545 (1 mg/mL) in 100 mM PBS pH 7.4. Excitation at 980 nm. **(B)** The overlap of the absorption bands of GO (10 μM) and GR with the emission bands of PTIR475 (1 mg/mL). Excitation at 980 nm. All spectra were recorded in 0.1 M sodium acetate buffer pH 5.1.

Four proteins possessing surface exposed cysteine residues and suitable co-factors for energy transfer studies were selected for covalent attachment to the surface of the UCPs. These are the truncated heme domain of cytochrome P450 BM3/CYP102A1 (BM3Heme), cytochrome *c* (cytC), glucose oxidase (GO) and glutathione reductase (GR). Figure 4 shows the overlap for the absorption spectra of each of these biomolecules with the emission from the UCPs: BM3Heme and cytC with the Er(III)-centered emission at 545 nm, and GO and GR with the Tm(III)-centered emission at 475 nm.

BM3Heme and cytC are heme proteins whose function depends upon the iron-porphyrin complex within the active site (Paoli et al., 2002). P450s enzymes such as BM3 are a family of enzymes that catalyse the oxygenation of a number of substrates (Girvan et al., 2007) and are responsible for the metabolism of drugs within the body (Ogu and Maxa, 2000). cytC is a small (12 kDa) redox active protein that is important for life (Hüttemann et al., 2011). It is the final electron carrier in the electron transport chain of the mitochondria during respiration (Hüttemann et al., 2011). It is also an important component in apoptosis (cell death). The electron transport capabilities of cytC lies in its heme cofactor, which is bound to the enzyme by two thioether bonds to cysteine residues from the protein (Bertini et al., 2006; Hüttemann et al., 2011).

GO and GR are flavoenzymes from a group of enzymes that catalyse the oxidation of a variety of different substrates (Walsh and Wencewicz, 2013). They contain either FMN or FAD (flavin adenine dinucleotide) redox active cofactor. GO is a FAD containing enzyme that catalyses the oxidation of β-D-glucose to β-D-glucono-1,5-lactone and H_2_O_2_ (Gibson et al., 1964). GO is most commonly used as an electrochemical blood glucose sensor for monitoring/diagnosing diseases such as diabetes (Heller and Feldman, 2008; Harper and Anderson, 2010; Bruen et al., 2017). GR is an NADPH dependent enzyme containing a FAD cofactor (Mavis and Stellwagen, 1968; Mittl and Schulz, 1994) that catalyses the reduction of oxidized glutathione disulphide (GSSG) to reduced glutathione (GSH) (Nimse and Pal, 2015). Reduced glutathione is used as an antioxidant in human tissues and can act as a biomarker for diseases such as Parkinson's, Alzheimer's, diabetes and HIV (Timur et al., 2008; Liu et al., 2017).

**Scheme 2:**
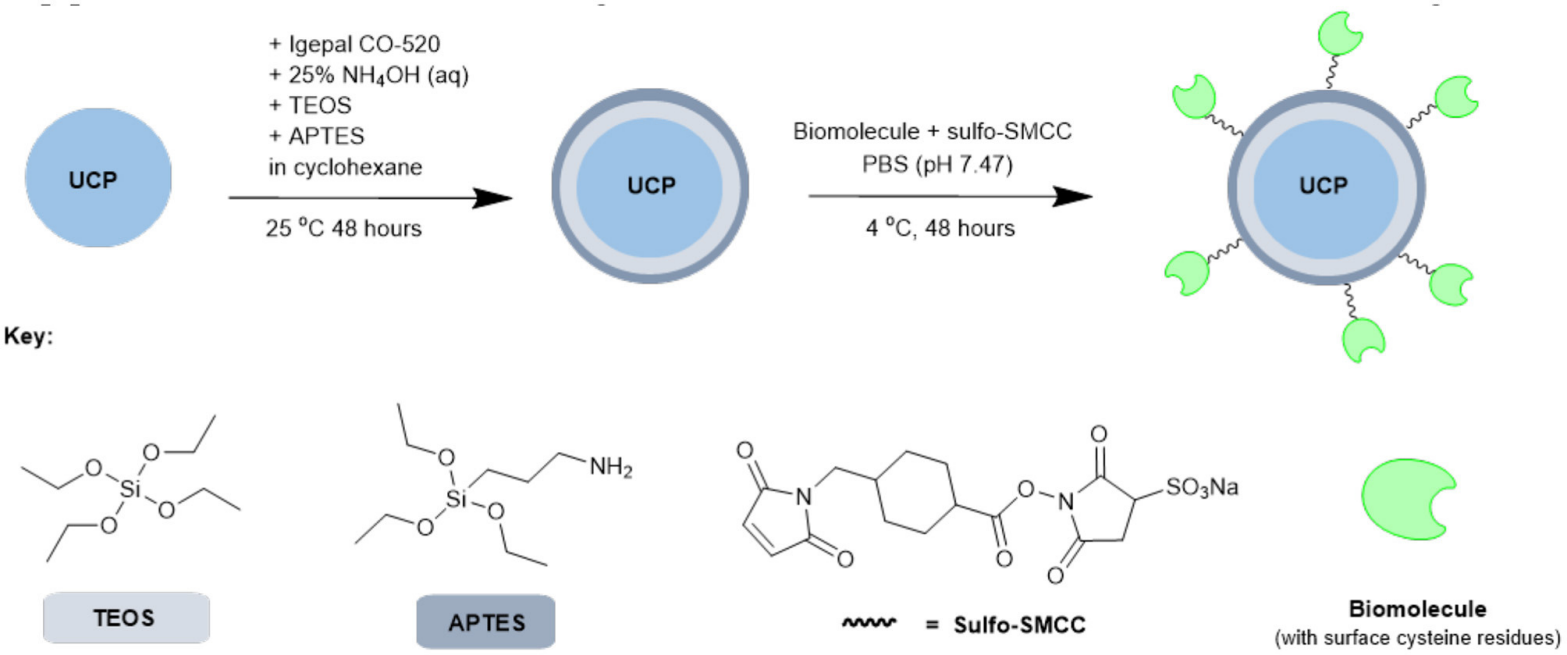
Simplified representation of the overall synthetic scheme for conjugation of biomolecules (or similar) to UCPs.

Based on the results from our screening studies with GFP, these 4 proteins/enzymes were all attached to UCPs using the method illustrated in Scheme 2. PTIR475 and PTIR545 UCPs were first capped with silica using a reverse microemulsion synthesis, with IGEPAL® CO-520 used to stabilize the procedure during the polymerization of tetraethyl orthosilicate (TEOS) (Muhr et al., 2014). The silica layer is important as it protects that UCP surface against solution quenching processes, it adds biocompatibility and provides the ability to easily functionalize further. In addition to TEOS (3-aminopropyl)triethoxysilane (APTES), was added to the reaction mixture to produce an accessible surface layer of primary amines within the silica shell.

The resulting APTES-coated UCPs can be coupled to the proteins with surface cysteines using a sulfo-SMCC cross-linker. This linker contains both an NHS ester and a maleimide and was first allowed to couple (via maleimide-cysteine conjugation) to the protein before the addition of APTES545 or APTES475 (via NHS-ester-amine conjugation). The reaction mixture was gently agitated under mild conditions to allow the coupling to progress and after each stage of this multi-step procedure the UCPs were centrifuged and washed several times to remove unreacted reagents.

**Figure 5:**
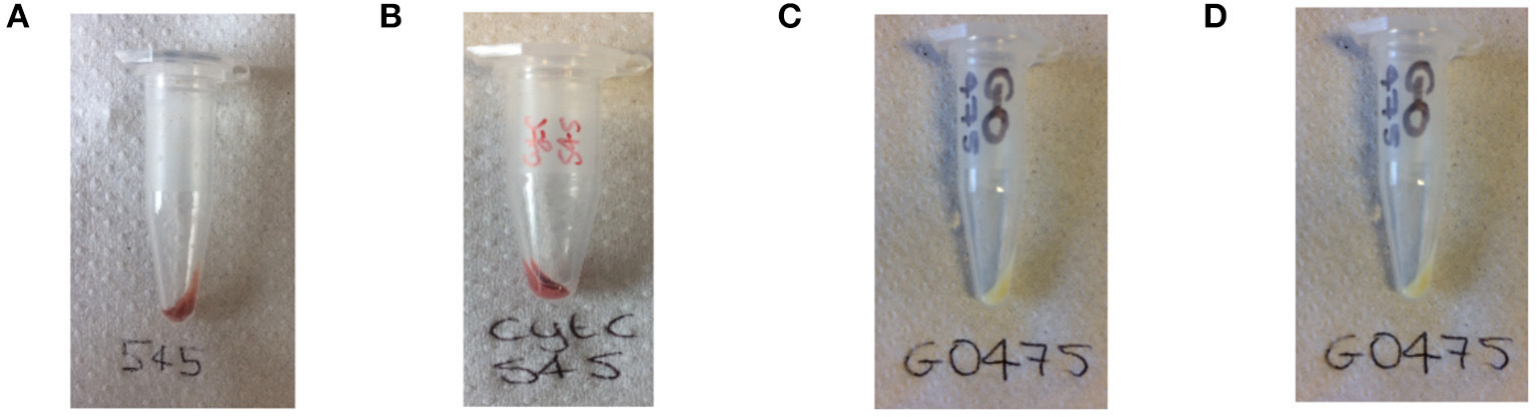
Photograph of the: **(A)** BM3Heme545 product obtained from the covalent attachment of BM3Heme to APTES545, **(B)** cytC545 product obtained from the covalent attachment of cytC to APTES545, **(C)** GO475 product obtained from the covalent attachment of GO to APTES475, **(D)** GR475 product obtained from the covalent attachment of GR to APTES475.

As with dye-UCP and GFP-UCP conjugation reactions, visible color changes were an initial indication of reaction success (Figure 5). Again, the pellets were washed until no free biomolecule (protein or cofactor) could be observed in the supernatants by UV-vis absorption spectroscopy (Supplementary Figure 22). Note that the presence of biomolecule absorption in the first of these washes indicates that they were added in suitable excess in all cases. The reactions with BM3Heme and cytC produced pellets with a strong red color, and with GO, a yellow pellet was obtained. The reaction with GR did not result in an observable color change by eye, but it should be noted that the quantity of enzyme in this reaction was far smaller due to availability of the stock suspension. Therefore, it is quite possible that the concentration of GR conjugated is simply too low to be seen with the naked eye.

**Figure 6:**
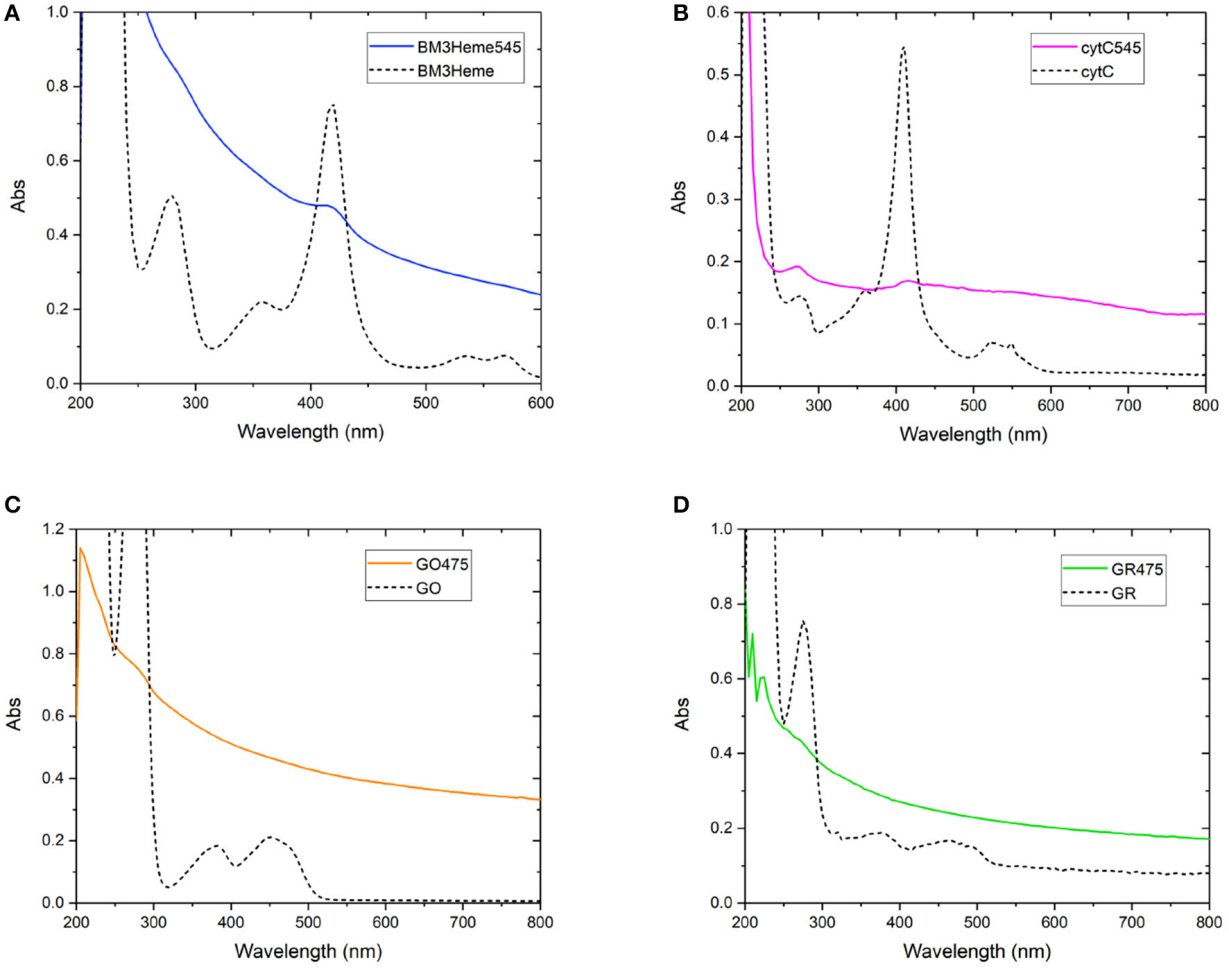
Solution UV-vis spectra of biomolecule-UCP conjugates (solid lines), overlaid with the UV-vis spectrum of each relevant biomolecule (dashed lines): **(A)** BM3Heme545 and BM3Heme (10 mM PBS, pH 7.4), **(B)** cytC545 and cytC (100 mM PBS pH 7.4), **(C)** GO475 and GO (100 mM PBS, pH 7.4), and **(D)** GR475 and GR (10 mM PBS pH 7.4).

As with the initial organic dye studies, UV-vis spectra (Figure 6) were dominated by scattering from the UCP dispersion. BM3Heme545 (Figure 6A) did, however, show a distinct peak at 418 nm corresponding to the absorption maximum of BM3Heme (Munro et al., 2007). Furthermore, cytC545 exhibited small peaks at 280 and 410 nm, which do align with the absorption peaks of cytC, albeit with different relative magnitudes. Reflectance measurements were also conducted (Supplementary Figure 27), and also show features centered at 280 nm from BM3Heme545, cytC545, and GR475, which is likely to originate from the aromatic residues in these proteins.

**Figure 7:**
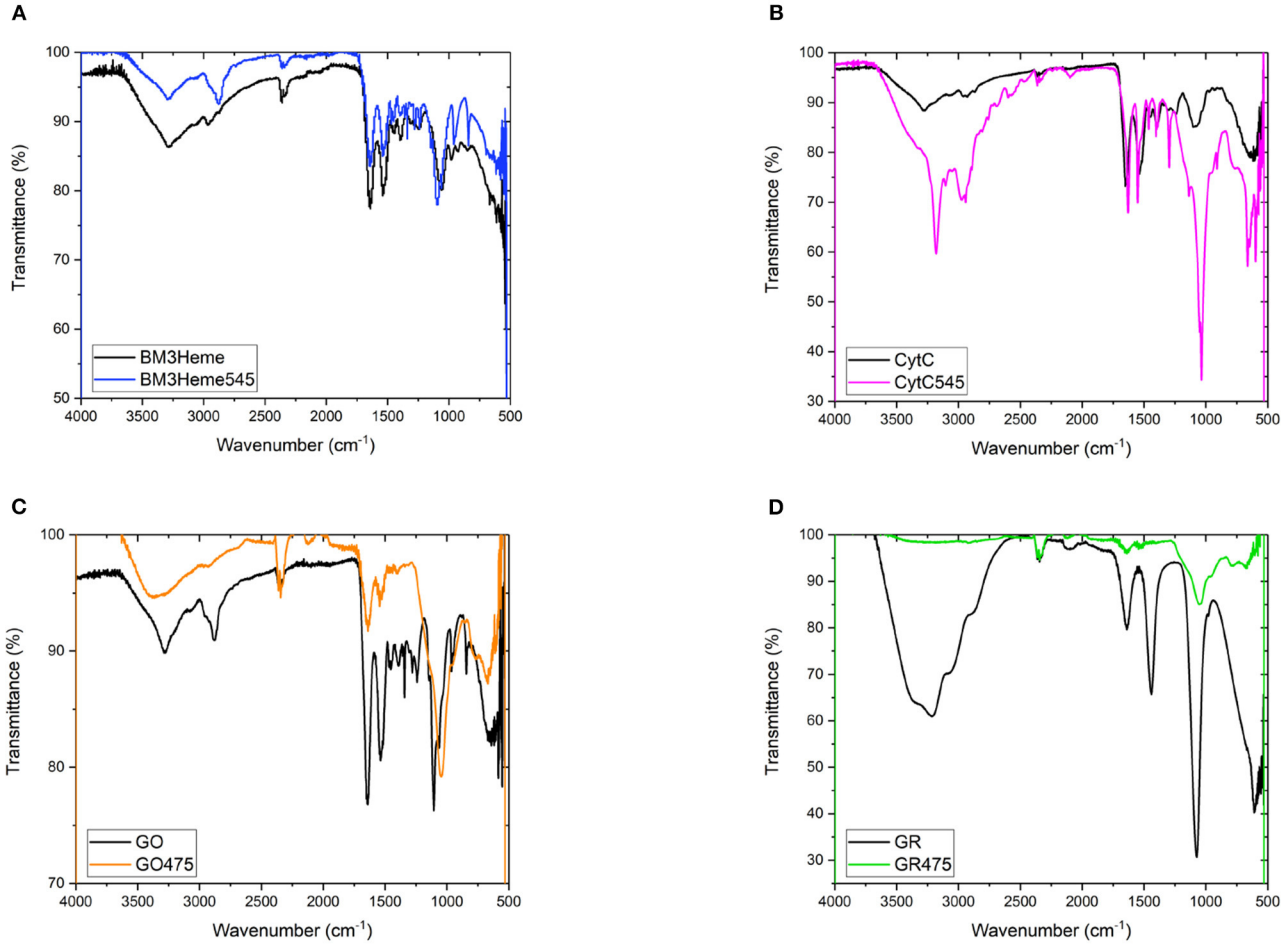
**(A)** The FTIR spectra of BM3Heme and BM3Heme545, **(B)** the FTIR spectra of CytC and cytC545, **(C)** the FTIR spectra of GO and GO475, and **(D)** the FTIR spectra of GR and GO475.

The IR spectra for all bioconjugates show the characteristic profiles of proteins, with the amide (NH, CO), carboxylic acid and CH fundamental stretching modes apparent, consistent with the presence of BM3Heme, cytC, GO, and GR on the surface of the UCPs (Figure 7). The GR475 spectrum exhibits weaker intensity than the other protein-UCP conjugates. This suggests, as indicated by visual inspection, that there is a low concentration of protein present in this system.

**Figure 8:**
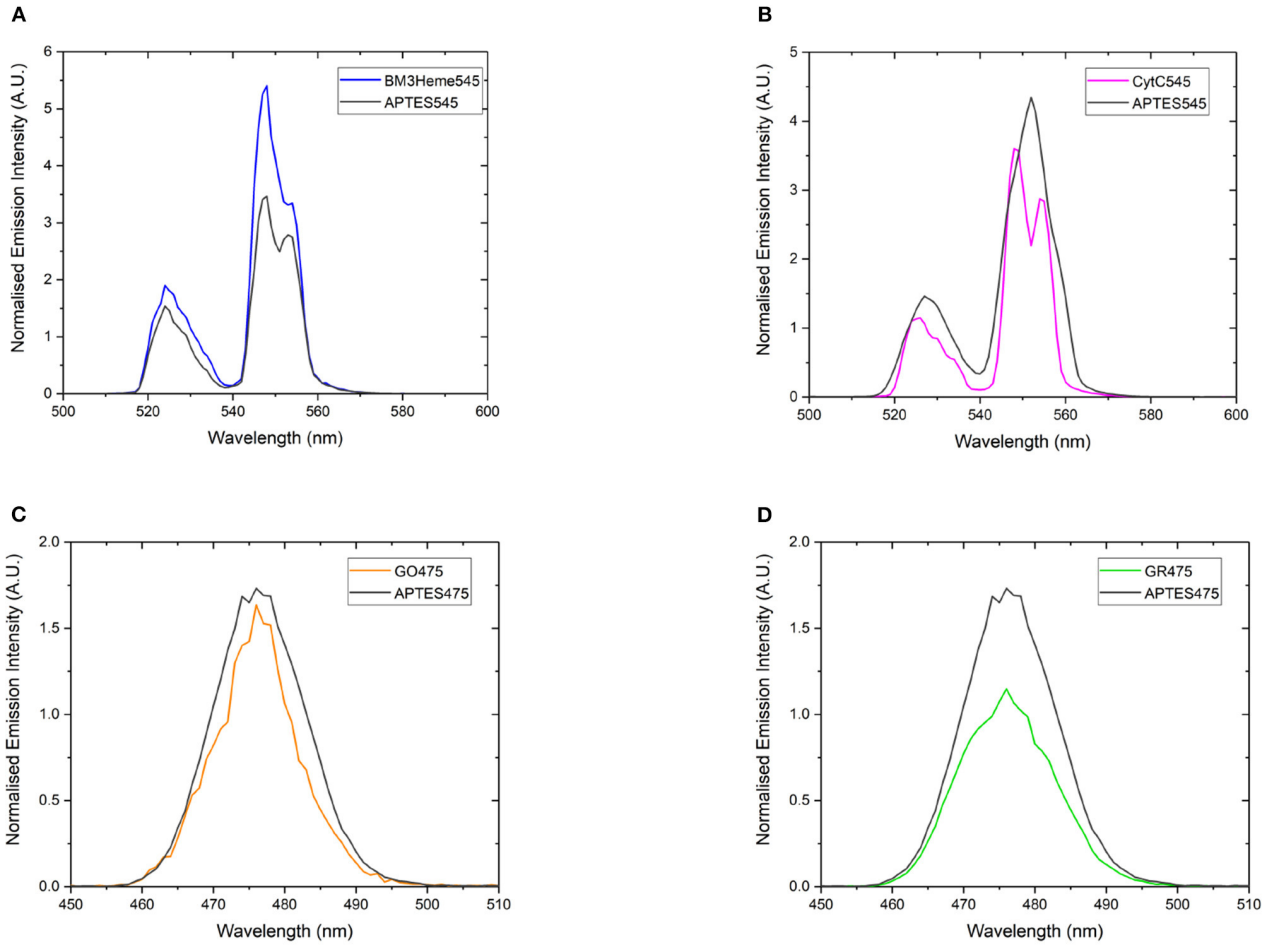
The normalized emission spectra of: **(A)** APTES545 and BM3Heme545 showing only the 545 nm band, **(B)** APTES545 and cytC545 showing only the 545 nm band, **(C)** APTES475 and GO475 showing only the 475 nm band, **(D)** APTES475 and GR475 showing only the 475 nm band. All samples were 1 mg/mL in in PBS buffer (100 mM, pH 7.4). All spectra have been recorded following excitation at 980 nm and are reported without correcting for the detector response. **(A,B)** Have been normalized to the 660 nm band, and **(C,D)** have been normalized to the 800 nm band (not shown here, see Supplementary Figure 25 for expanded spectra).

If the protein has successfully been attached to the surface of the UCPs, it is expected that a decrease in the emission intensity of the UCP emission bands will be observed due to AET from the UCP to the protein cofactor (heme or flavin). Therefore, a comparison of the emission profiles of the UCP-biomolecule to the precursor UCPs can be used to determine if the intact protein is present on the surface of the UCPs. The expected decrease in emission intensity was observed for cytC545, GO475, and GR475 (Figures 8B–D). The same was not found for BM3Heme545 (Figure 8A), which instead shows an increased emission; the reason for which remains unclear, but may indicate that this protein is acting as an extra hydrophobic layer preventing extra quenching of the UCPs with the surrounding water molecules and/or that surface coverage of the UCP has changed during manipulation. However, the data generally support a successful attachment of proteins/enzymes.

The analysis of the UV-vis, IR, fluorescence and color change of the UCPs, strongly suggests that the method described can be used to attach a range of proteins and enzymes that possess a surface/solvent-exposed cysteine residue. We have demonstrated the versatility of this method, having attached four different proteins; BM3Heme, cytC, GO, and GR (in addition to GFP). However, in order for future applications of UCP-protein systems as biosensors, it is necessary to prove that activity is retained upon conjugation.

**Figure 9:**
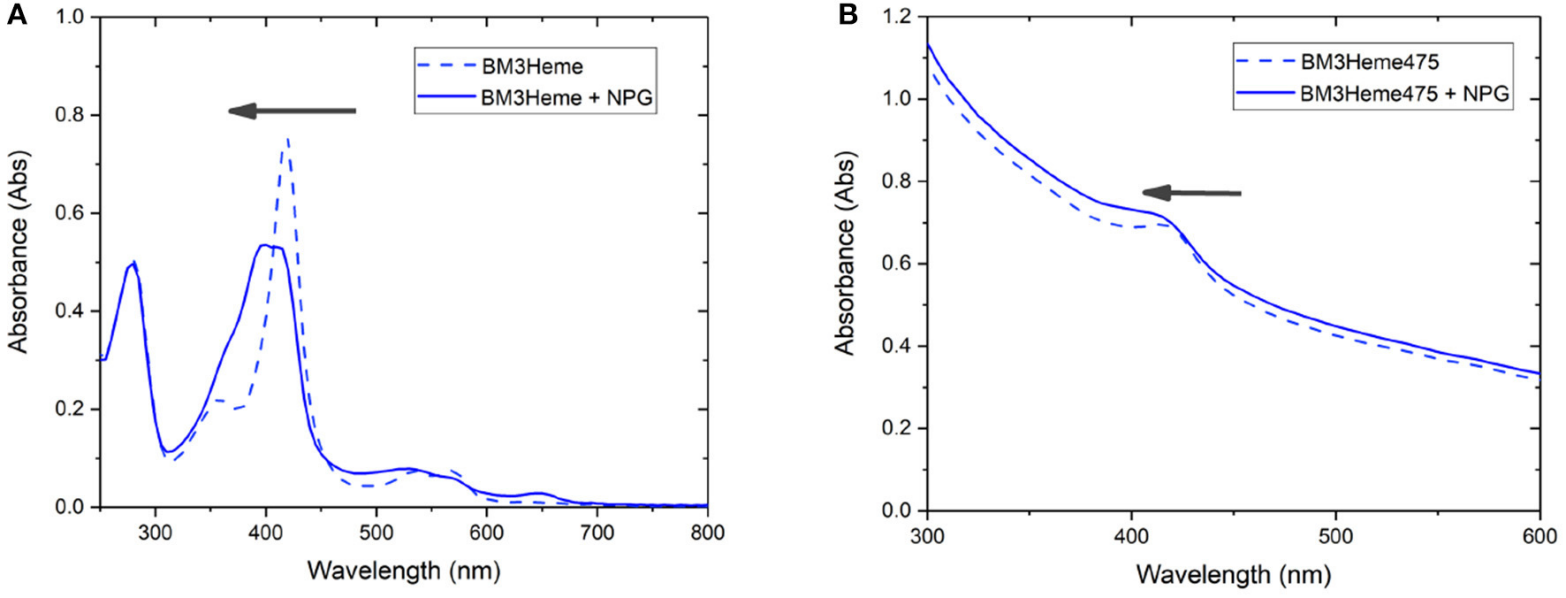
The UV-vis spectrum of: **(A)** BM3Heme (10 μM in 100 mM PBS, pH 7.4) before and after the addition of NPG (10 μM) **(B)** BM3Heme545 (1 mg/mL in 100 mM PBS pH 7.4) before and after the addition of 20 μM NPG (20 μM).

Cytochrome P450 catalyses the oxidation of a variety of substrates within the body such as *N*-palmitoylglycine (NPG) (Zhang et al., 2020). Within the enzyme, the BM3Heme domain is responsible for substrate binding but requires the partner redox domain in order to be catalytically competent. Substrate binding causes a change in the relative intensities of the absorption bands (Figure 9A). This 10 nm shift in the absorption band of BM3Heme upon binding to NPG is also observed in the UV-vis spectrum of BM3Heme545 (Figure 9B) suggesting that the BM3Heme bound to the surface of the UCP has maintained its ability to bind to substrate in a way that is similar to the protein free in solution. Like BM3Heme, the UV-vis spectrum of cytC differs depending on whether the enzyme is in its oxidized or reduced form (Supplementary Figure 24). However, as we were unable to obtain a reliable UV-vis spectrum of cytC545 we were unable to repeat this experiment to see if cytC had maintained its activity when bound to the UCP surface.

**Figure 10:**
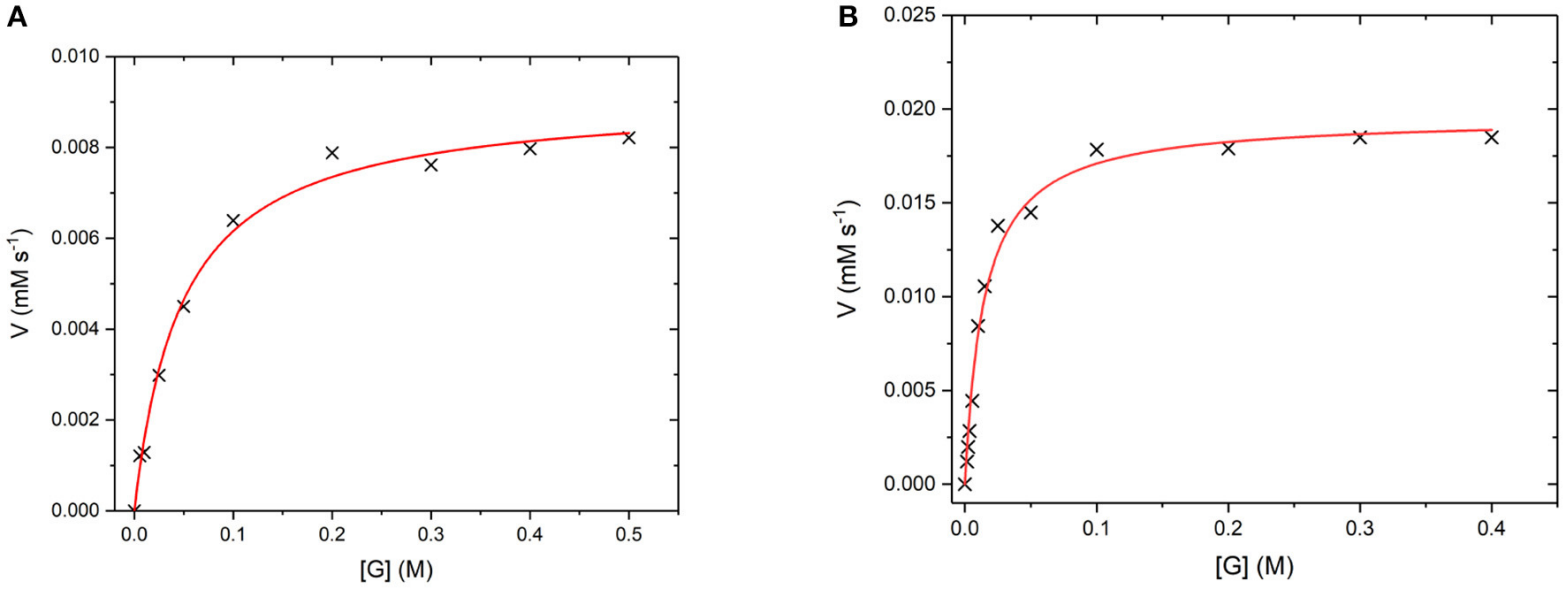
**(A)** Michaelis-Menten curves for the reaction of **(A)** GO (8 nM) and **(B)** GO475 (1 mg/mL) with D-glucose (G) as the substrate and 2.3 mM benzoquinone as the electron acceptor. Reactions were performed in degassed 0.1 M sodium acetate buffer at pH 5.1 by monitoring the formation of reduced benzoquinone (hydroquinone) at 290 nm using ε = 2.31 mM^−1^ cm^−1^. Red lines show fits to the Michaelis-Menten equation and fitted parameters are given in the main text.

GO is an FAD containing enzyme that catalyses the oxidation of β -D-glucose to β -D-glucono-1,5-lactone and H_2_O_2_ (Gibson et al., 1964). During the reaction, the cofactor FAD accepts an electron from the glucose substrate resulting in reduced GO. Then oxygen or an artificial oxidant acts as an electron acceptor, accepting the electron from reduced GO, resulting in oxidized GO. This oxygen then reduces to produce the H_2_O_2_ by-product (Leskovac et al., 2005). In order to determine if GO has retained its catalytic ability when covalently bound to the UCP surface, the steady state turnover and kinetics were measured. Figure 10 below shows a Michaelis-Menten plot for the D-glucose concentration dependence of the activity of free GO and for GO475. Both GO and GO475 show typical Michaelis-Menten (saturation) behavior when using D-glucose as the limiting substrate and an excess of benzoquinone as the electron acceptor. These data were fitted to the Michaelis-Menten equation:

$$\begin{array}{l} V_{obs}=\frac{V_{\text{max}}\left[S\right]}{K_{M}+\left[S\right]};\;V_{\text{max}}=k_{cat}{\left[E\right]}_{0} \end{array}$$

giving a *k*_cat_ value of 1,139 ± 29 s^−1^ and a *K*_M_ value of 49.5 ± 4.8 mM for GO, which is close to the literature value of *K*_M_ = 33–110 mM (Gibson et al., 1964). A *K*_M_ of 14.5 ± 1.4 mM and *V*_max_ of 19.5 ± 0.5 μM s^−1^ were found for 1 mg/mL GO475. The difference in *K*_m_ values suggest changes to the kinetics of glucose binding and/or dissociation perhaps through some modest alteration or occlusion of the enzyme active site. Nevertheless, the *K*_M_ values for the GO475 system is of the same order of magnitude as the free GO, suggesting that there is an appreciable quantity of GO bound to the UCP that has retained its catalytic function after covalent attachment.

**Figure 11:**
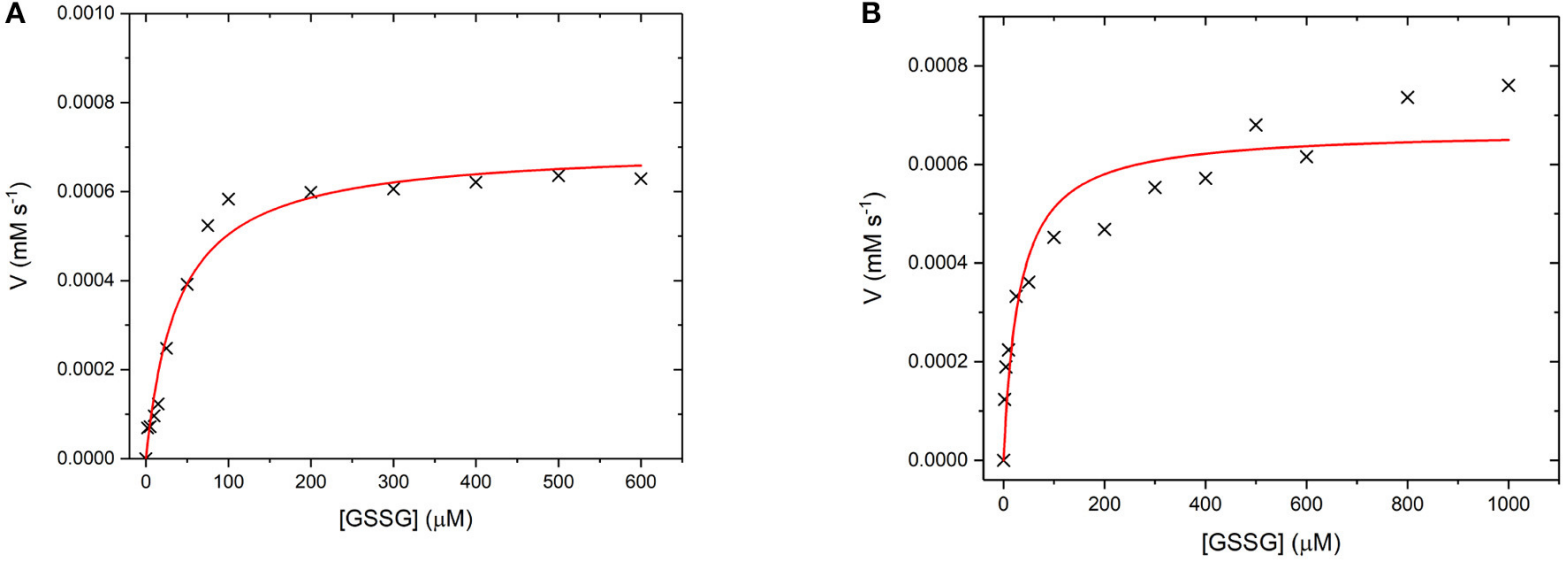
**(A)** Michaelis-Menten curve for the enzyme turnover of **(A)** GR (10 nM) and **(B)** GR475 (1 mg/mL) with 93 μM NADPH and varying GSSG. Reactions were performed in degassed potassium phosphate buffer, pH 7 by monitoring the depleting of NADPH at 340 nm using ε = 6.022 mM^−1^ cm^−1^. Red lines show fits to the Michaelis-Menten equation and fitted parameters are given in the main text.

GR is an NADPH dependent, FAD containing enzyme that catalyses the reduction of oxidized glutathione disulfide (GSSG) to reduced glutathione (Nimse and Pal, 2015). The first step is the reduction of the FAD by NADPH before binding of the substrate GSSG in the active site. The enzyme then catalyses the reduction of the disulfide bond in GSSG to produce two glutathione molecules and the re-oxidized GR enzyme (Nimse and Pal, 2015). Again, to assess whether GR has retained its catalytic ability when covalently bound to the UCP surface, the enzyme kinetics of GR and GR475 were measured (Figure 11). Both GR and GR475 show typical Michaelis-Menten type behavior with the oxidizing substrate GSSG when NADPH consumption is measured. A *k*_cat_ value of 70 ± 3 s^−1^ and *K*_M_ value of 39.4 ± 6.2 μM was obtained for GR, with the *K*_M_ similar to the literature value of 53.1 ± 3.4 μM (Can et al., 2010). 1 mg/ml GR475 gives a *K*_M_ value of 30.9 ± 9.6 μM, which is not significantly different to that measured for free GR and a *V*_max_ value of 0.67 ± 0.01 μM s^−1^. These data suggest that binding of GR to the UCP is successful and the enzyme maintains its activity.

## Conclusion

In summary, we have demonstrated that a variety of different conjugation methods can be used to covalently attach a wide variety of different molecules including organic dyes, enzymes and proteins to the surface of UCPs. Several thiol-thiol and thiol-maleimide coupling methods were screened for the attachment of enhanced GFP as a model protein with surface exposed cysteine residue to the surface of a range of UCPs (80 nm to 1.5 μM in diameter). One particularly successful method was identified and demonstrated to be a robust and versatile method for the covalent attachment of proteins containing an accessible surface cysteine group to the surface of upconverting phosphors. This method was used to attach the proteins and enzymes: BM3Heme and cytochrome C to YbEr doped UCPs (PTIR-545), and glucose oxidase and glutathione reductase to YbTm doped UCPs (PTIR-475). The significance of this conjugation method is that the proteins remain catalytically active when coupled to the UCP surface. This opens up the opportunity to develop UCP-biomolecule biosensors with increased sensitivity due to the close proximity (within AET distance) of the UCP donor and biomolecule acceptor, particularly with smaller UCNPs where Förster resonance energy transfer of the majority of emitting ions within the entirety of the nanoparticle can be exploited. Studies toward this (to enhance the luminescence response) are in progress.

## Data Availability Statement

The original contributions presented in the study are included in the article/Supplementary Materials further inquiries can be directed to the corresponding author/s.

## Author Contributions

LB, AJ, SH, and LN conceived and designed the experiments. LB performed the experiments and resulting analysis, with input from HW. LB and HW wrote the manuscript with input from all authors. All authors contributed to the article and approved the submitted version.

## Conflict of Interest

The authors declare that the research was conducted in the absence of any commercial or financial relationships that could be construed as a potential conflict of interest.
