# Supplementary material for: Assessing the Covalent Attachment and Energy Transfer Capabilities of Upconverting Phosphors With Cofactor Containing Bioactive Enzymes
Source: Front Chem. 2020 Dec 21;8:613334. doi: 10.3389/fchem.2020.613334 (PMC7779683; doi:10.3389/fchem.2020.613334)

Supporting Information

Methods for the covalent attachment of organic dyes to UCPs:

Fluorescein isothiocyanate (FITC) attachment to APTES475 or APTES545
Rhodamine B isothiocyanate (RBITC) attachment to APTES475 or APTES545:

APTES475 or APTES545 (5 mg) suspended in deionised water (1 mL), was diluted using a sodium bicarbonate buffer (1 mL, pH 8.5). FITC (10 mg) or RBITC (10 mg) was dissolved in DMF (1mL). 100 µL of this dye stock was added to the UCP dispersion with constant stirring. The reaction mixture was stirred at room temperature in a sealed flask for 24 hours. The product was collected by centrifugation (4000 rpm, 10 minutes) and washed with DMF until the supernatant was clean.

Methyl Red (MR) attachment to silica475 or silica545:
APTES-MR was first dissolved in DMF at a concentration of 15 mg/mL. 200 µL of the stock
APTES-MR was added to a suspension of silica475 or silica545 (5 mg/mL, aqueous). The reaction mixture was stirred at room temperature in a sealed flask for 24 hours. The product was obtained by centrifugation (4000 rpm, 10 minutes) and washed with DMF until the supernatant was clean

SAMSA fluorescein (SF) attachment to APTES475 or APTES545:
SF (5 mg) was first activated with 0.1 M NaOH (500 µL). After 15 minutes, the solution was neutralized with 1 M HCl (42 µL) and sodium phosphate buffer (0.1 mL, 0.5 M, pH 7) was added. APTES475 or APTES545 (5 mg/mL) aqueous suspension was diluted using phosphate buffer (800 µL, 0.1 M, pH 7.4) before sulfo-SMCC (1 mg, 1.5 mM) and deionised water (200 µL) were added. After 15 minutes, activated SF (600 µL) was added to the suspension. The reaction mixture was stirred at room temperature in a sealed flask for 24 hours. The product was obtained by centrifugation (4000 rpm, 10 minutes) and washed with sodium acetate buffer (0.5 M, pH 7) until the supernatant was clean.

Fluorescein maleimide (FM) attachment to MPTMS475 or MPTMS545:
MPTMS475 (5 mg/mL, aqueous solution) was mixed with FM (500 µL, 0.005 M aqueous solution). The reaction mixture was stirred at room temperature in a sealed flask for 24 hours. The product was obtained by centrifugation (4000 rpm, 10 minutes) and washed with deionised water until the supernatant was clean.

Supplementary figures relating to the attachment of organic dyes (FITC, MR, RBITC, SF, FM) to PTIR475 and PTIR545

Figure S1	Structure of organic dyes
Figure S2	Spectral overlap of dyes with UCPs
Figure S3	Solution UV-vis spectra of the washes from dye-UCP reactions
Figure S4	Photograph of products
Figure S5	Solution UV-vis spectra
Figure S6	Solid state reflectance spectra
Figure S7	Emission spectra
Figure S8	FTIR spectra
Figure S9	Raman spectra
Figure S10	TGA data
Figure S11	DLS size, PDI and ZP data
Figure S12	TEM images

Supplementary figures relating to the attachment of GFP to a number of precursor UCPs

Figure S13	Solution UV-vis spectra of the washes from GFP-UCP reactions
Figure S14	Solution UV-vis spectra
Figure S15	Solid state reflectance spectra
Figure S16	Emission spectra
Figure S17	FTIR spectra
Figure S18	Raman spectra
Figure S19	TGA data
Figure S20	DLS size, PDI and ZP data
Figure S21	TEM images

Supplementary figures relating to the attachment of biomolecules (BM3Heme, CytC, GO, GR) to APTES475 or APTES545

Figure S22	Solution UV-vis spectra of the washes from biomolecule-UCP reactions
Figure S23	TEM images and size summary
Figure S24	Solution UV-vis spectrum of oxidized and reduced CytC
Figure S25	Emission spectra
Figure S26	DLS size, PDI and ZP data
Figure S27	Solid state reflectance spectra
Figure S28	Raman spectra
Figure S29	Emission spectra of silica545 and MPTMS545
Figure S30	Powder X-ray diffraction spectra of PTIR475 PTIR545

Supplementary figures regarding characterisation of PTIR-475 and PTIR-545 UCPs and precursor UCP systems prior to bioconjugation

Figure S30	Powder X-ray diffraction spectra of PTIR475 and PTIR545
Figure S31	IR and Raman spectra of MPS and MPS475
Figure S32	IR and Raman spectra of 6-aminohexanoic acid, 6-

maleimidohexanoic acid, oleic acid and AHAMHAOAYbTm

Figure S33 IR and Raman spectra of 6-maleimidohexanoic acid and MHAYbTm

Figure S34 IR and Raman spectra of polyethylenimine acid and PEIYbTm

Figure S35 IR and Raman spectra of DMSA and DMSAYbTm

Figure S36 IR and Raman spectra of cysteine and cysteineYbTm

Figure S37 IR and Raman spectra of MPTMS, MPTMS475 and MPTMS545

Figure S38 IR and Raman spectra of IR spectra of APTES475 and APTES545

Figure S1: Chemical structures of the organic dyes used: a) fluorescein isothiocyanate (FITC), b) methyl red (MR), c) rhodamine isothiocyanate (RBITC), d) SAMSA fluorescein (SF), e) fluorescein maleimide (FM).

a) b) d) e)

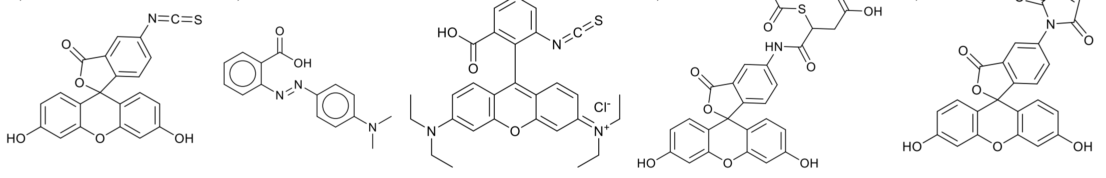

c)

Figure S2: Spectral overlap of the UV-vis absorption of organic dyes with UCP upconversion emission from PTIR475 and PTIR545. Dye spectra are normalized to their peak maximum, and UCP spectra to the maximum of their 475 and 545 nm peaks respectively. All spectra recorded in 100 mM TRIS pH 7.

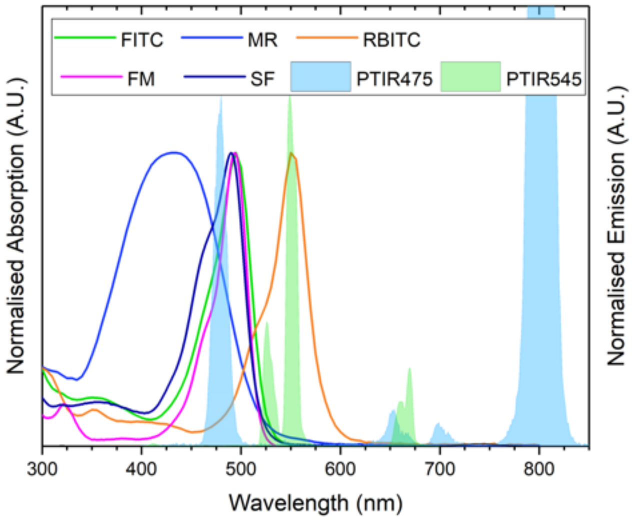

Figure S3: The UV-vis spectra of the washes from the dye attachment reactions with PTIR475 and PTIR545, all spectra were recorded in a 1cm^3 cuvette in PBS (100 mM, pH 7.4). a) FITC475, b) RBITC475, c) MR475, d) SF475, e) FM475 f) FITC545, g) RBITC545, h) MR545, i) SF545, j) FM545

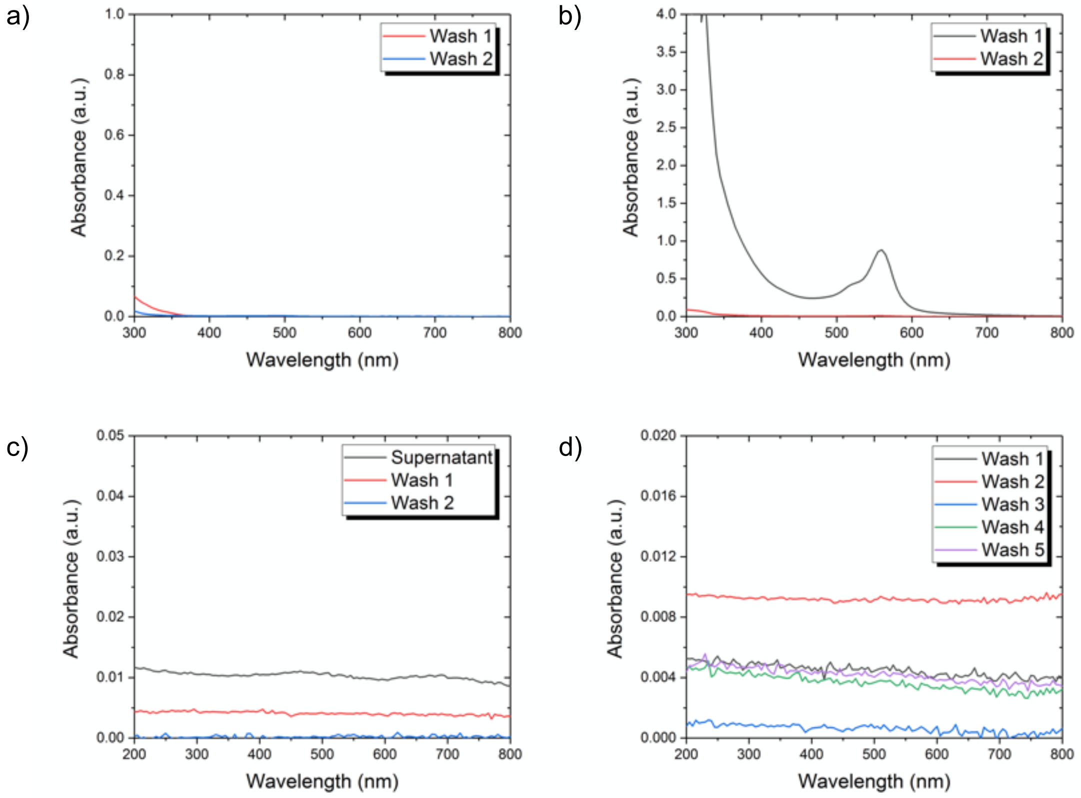

e)

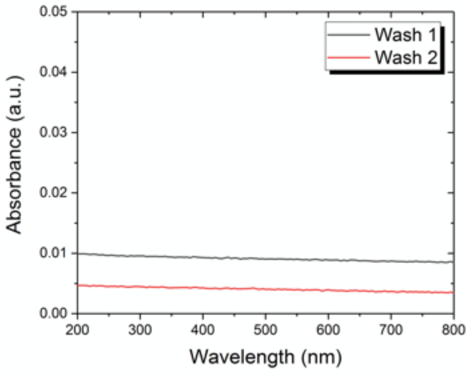

f)

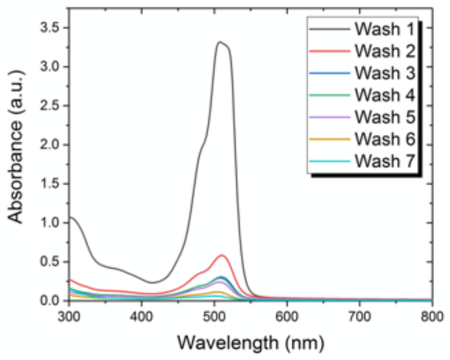

g)

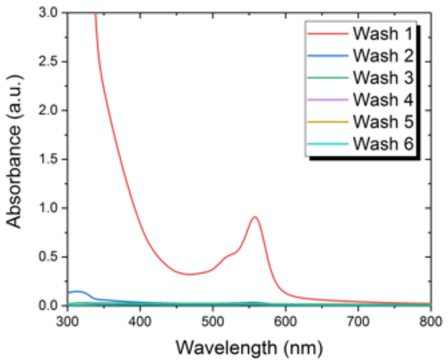

h)

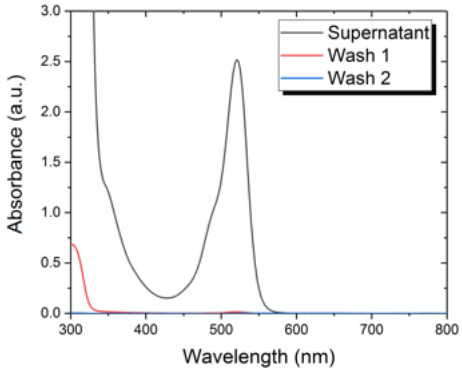

i)

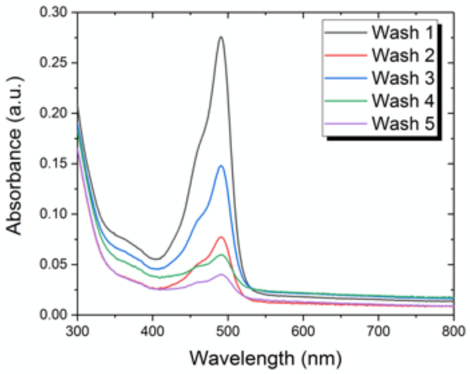

j)

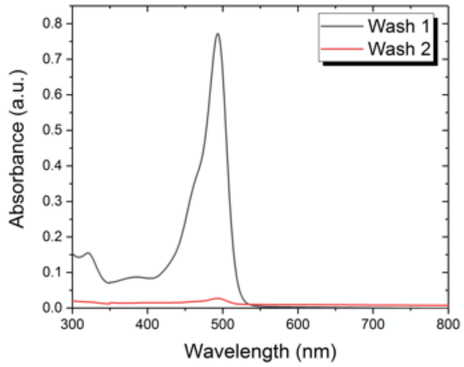

Figure S4: Photograph showing the products obtained from the covalent attachment of organic dyes to PTIR475. From left to right: SF475, FM475, RBITC475, FITC475 and MR475.

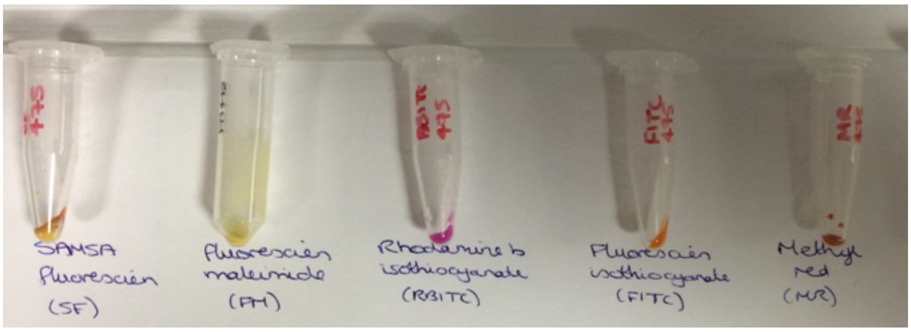

Figure S5: Solution UV-Vis spectra of (a) FITC475, RBITC475, MR475, SF475 and FM475 and b) FITC545, RBITC545, MR545, SF545 and FM545. All solutions were 1 mg/mL in 100 mM PBS pH 7.4.

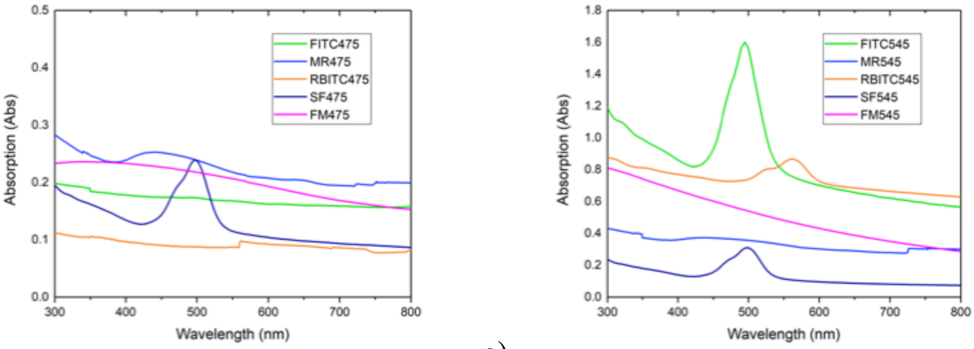

a) b)

Figure S6: Solid-state reflectance spectra of all dyes (solid line), DyePTIR475 conjugates (dashed line) and DyePTIR545 conjugates (dotted line). From left to right (top): FITC, RBITC, MR and (bottom) SF, FM.

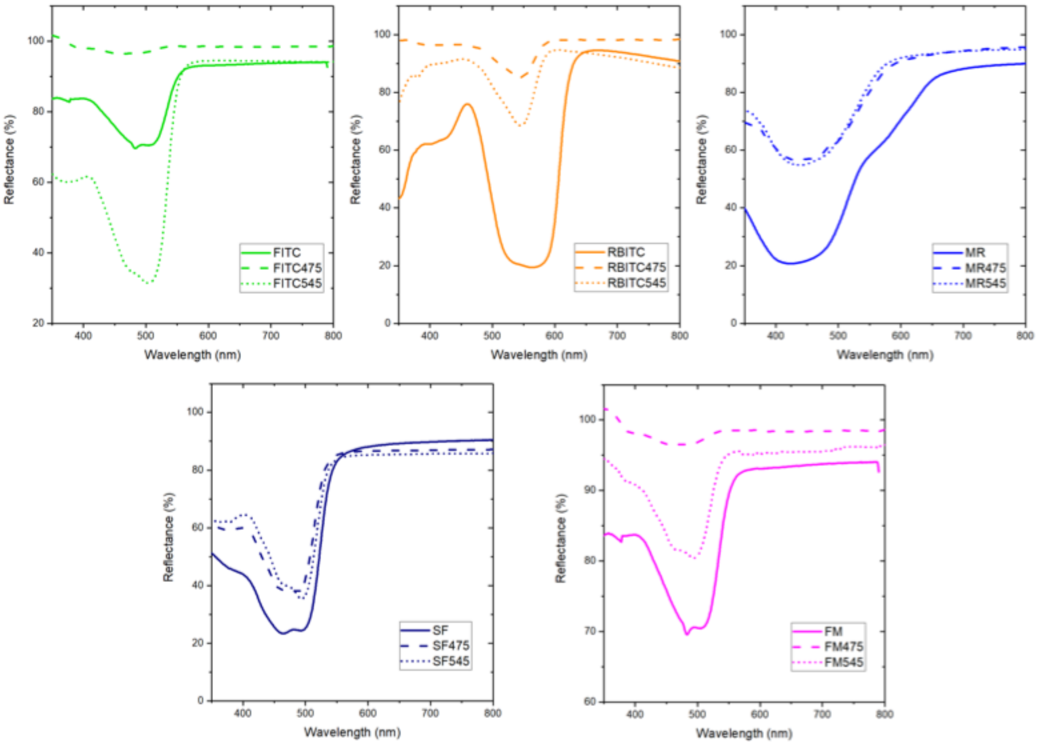

Figure S7: Normalised emission spectrum of a) APTES475, FITC475, RBITC475, MR475, SF475 and FM475 b) APTES545, FITC545, RBITC545, MR545, SF545 and FM545. All solutions were 1 mg/mL in 100 mM PBS pH 7.4. Excitation at 980 nm. All spectra are reported uncorrected for the detector response.

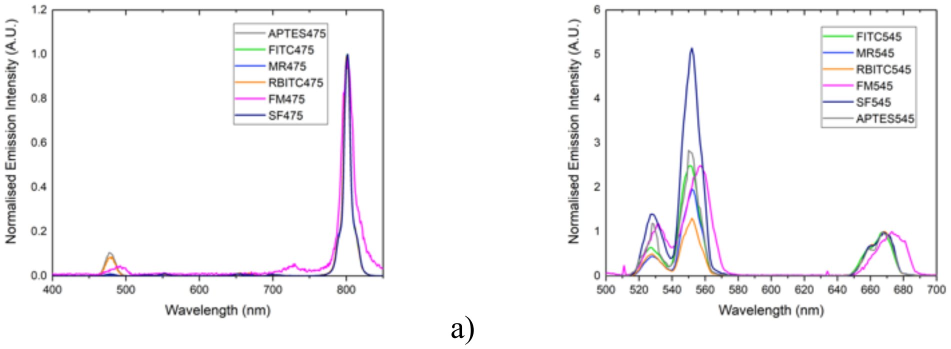

a) b)

Figure S8: FTIR spectra of a) FITC, FITC475 and FITC545, b) RBITC, RBITC475 and RBITC545, c), MR, MR475 and MR545, d) SF, SF475 and SF545, e) FM, FM475 and FM545.

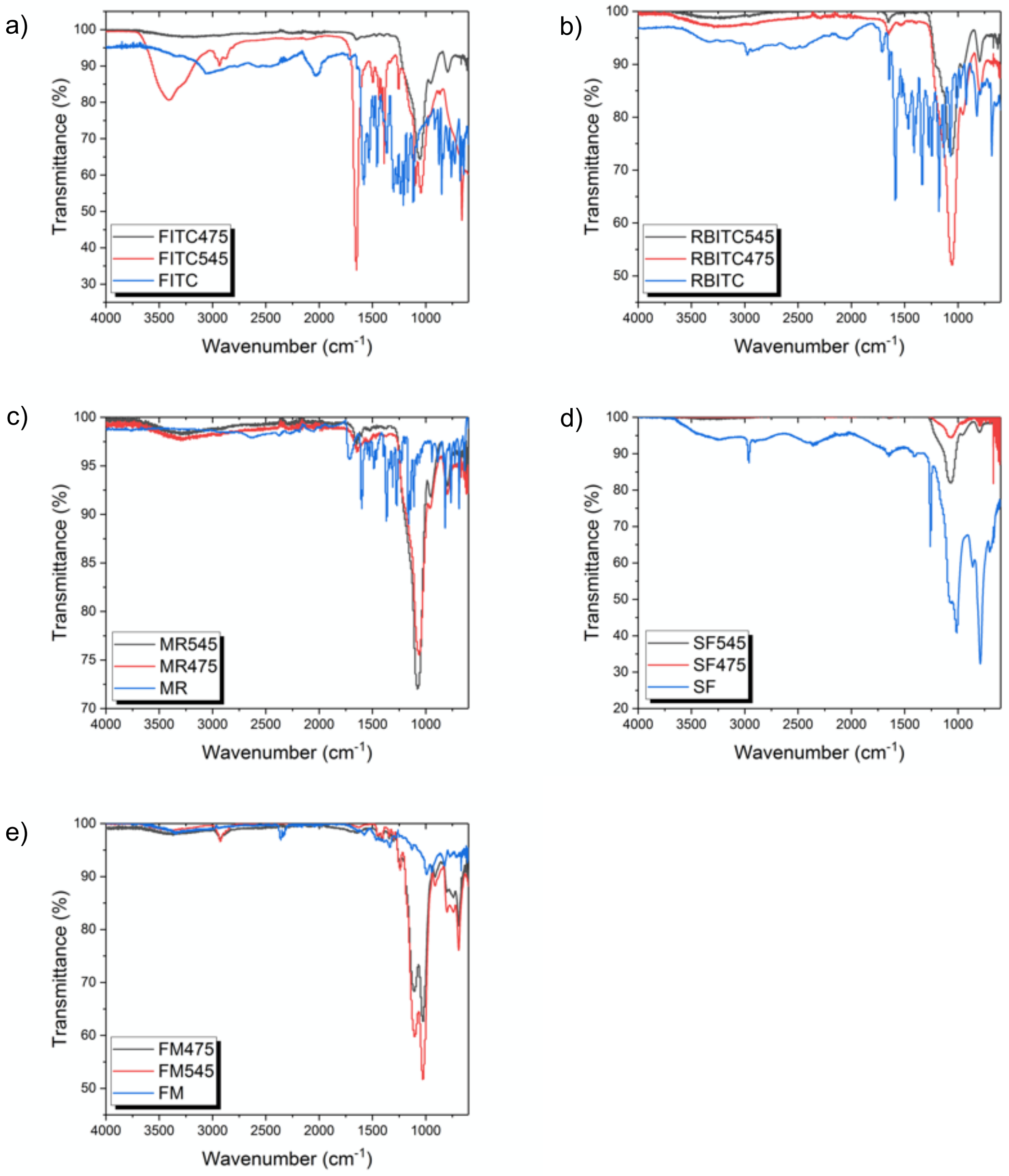

Figure S9: Raman spectra of a) FITC, FITC475 and FITC545, b) RBITC, RBITC475 and RBITC545, c), MR, MR475 and MR545, d) SF, SF475 and SF545, e) FM, FM475 and FM545.

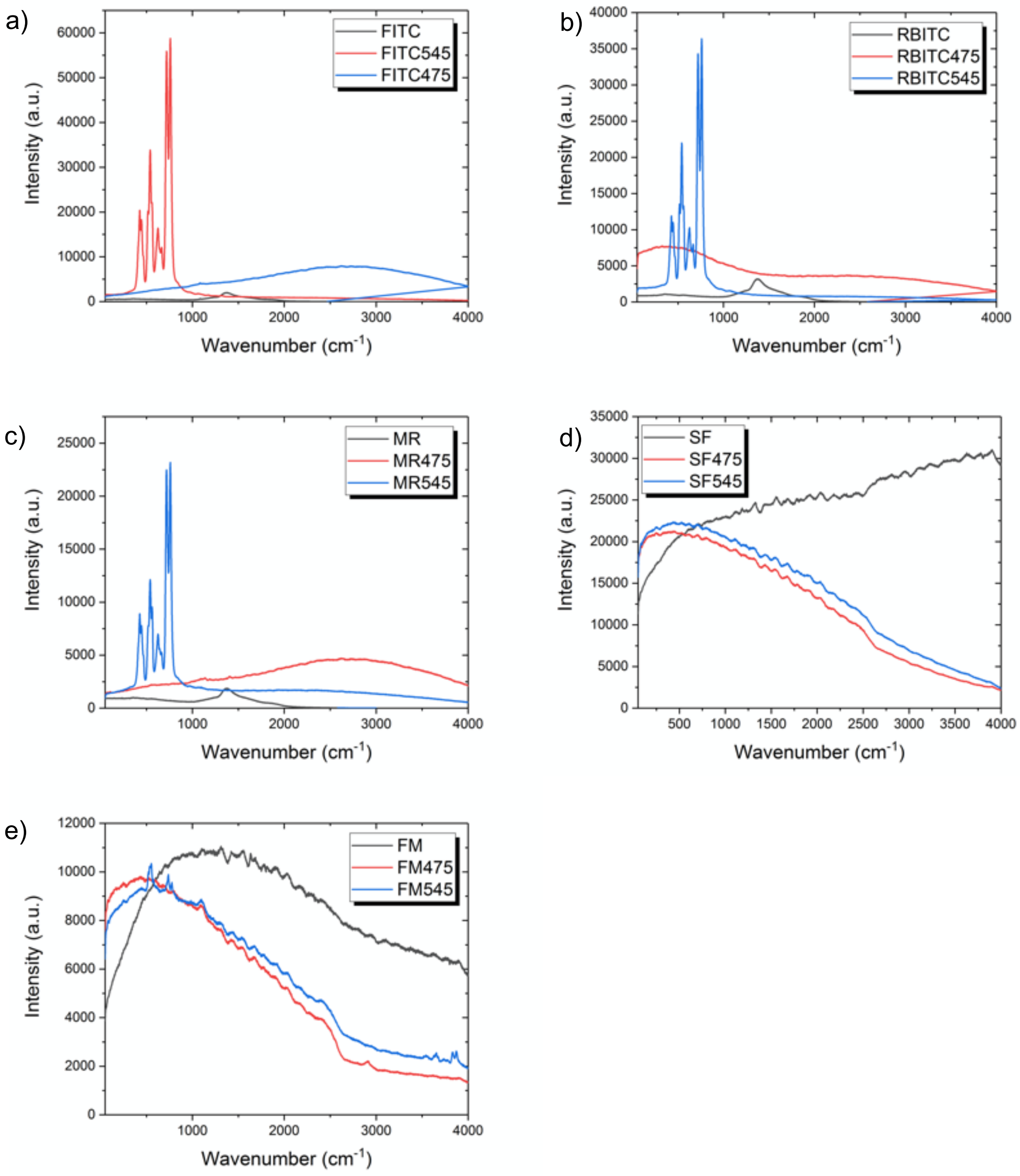

Figure S10: Summary of the TGA data for all the dye-UCP conjugates

	Step 1		Step 2		Step 3	
	Temperature (°C)	Weight loss (%)	Temperature (°C)	Weight loss (%)	Temperature (°C)	Weight loss (%)
FITC475	82.1	10.6	419	20.8		
FITC545	81.7	9.71	369	12.6	558	7.56
RBITC475	83.4	11.2	347	11.4	533	6.62

RBITC545	74.8	10.4	345	10.8	540	5.61
MR475	85.78	3.24	319.7	2.85	518.0	2.19
MR545	70.83	2.58	354.6	5.48	546.7	3.60
SF475	73.3	5.83	391	5.90		
SF545	72.2	4.16	344	5.40	510	1.35
FM475	354	37.6				
FM545	361	49.4				

Figure S11: Summary of size (z-average), polydispersity index (PDI) and zeta potential (ZP) for the GD_2SO_4:YbTm precursor phosphors and the dye conjugated products.

UCP system	z-average	PDI	ZP
Silca475	434	0.43	-34.73
APTES475	4964	0.767	-11.1
COOH475	2600	0.954	-29.4
MPTMS475	5284	0.363	-14.7
FITC475	6408	0.824	-27
RBITC475	8555	1.00	-17.13
MR475	2908	0.811	-6.24
SF475	3184	0.24	-30.03
FM475	1435	0.14	41.5

Figure S12: TEM images of: a) FITC475, b) RBITC475, c) MR475, d) SF475 and e) FM475.

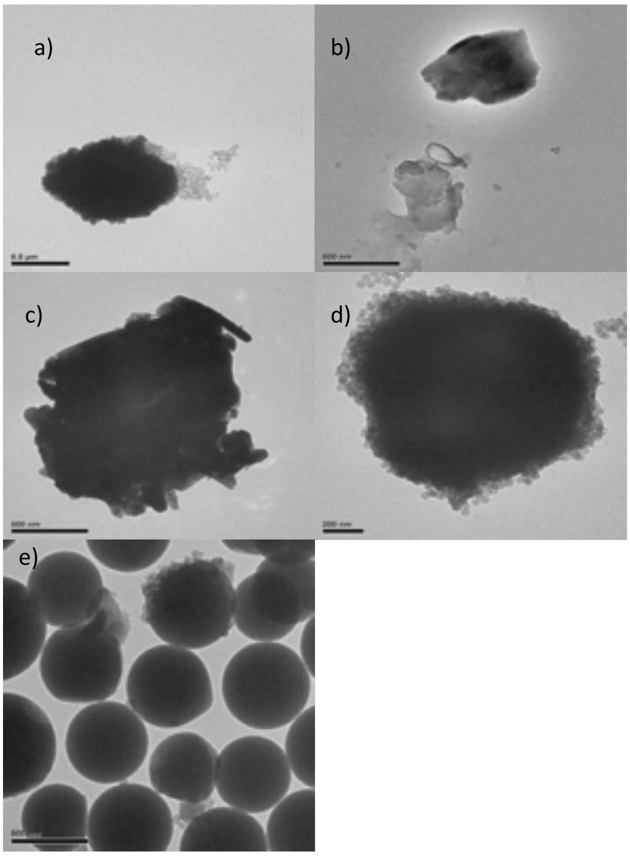

Figure S13: The UV-Vis spectra of the washes from the GFP attachment reactions, all spectra recorded in PBS (100 mM, pH 7.4). a) AHAMHAOAYbTm_GFP, b) APTES475_GFP, c) PEIYbTm_GFP, d) DMSAYbTm_GFP, e) MHAYbTm_GFP, f) CysteineYbTm_GFP, f) MPS475_GFP, g) MPTMS475_GFP.

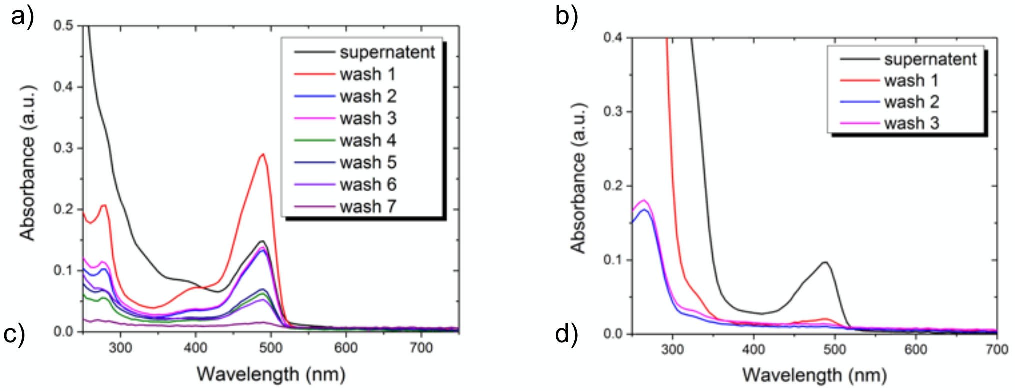

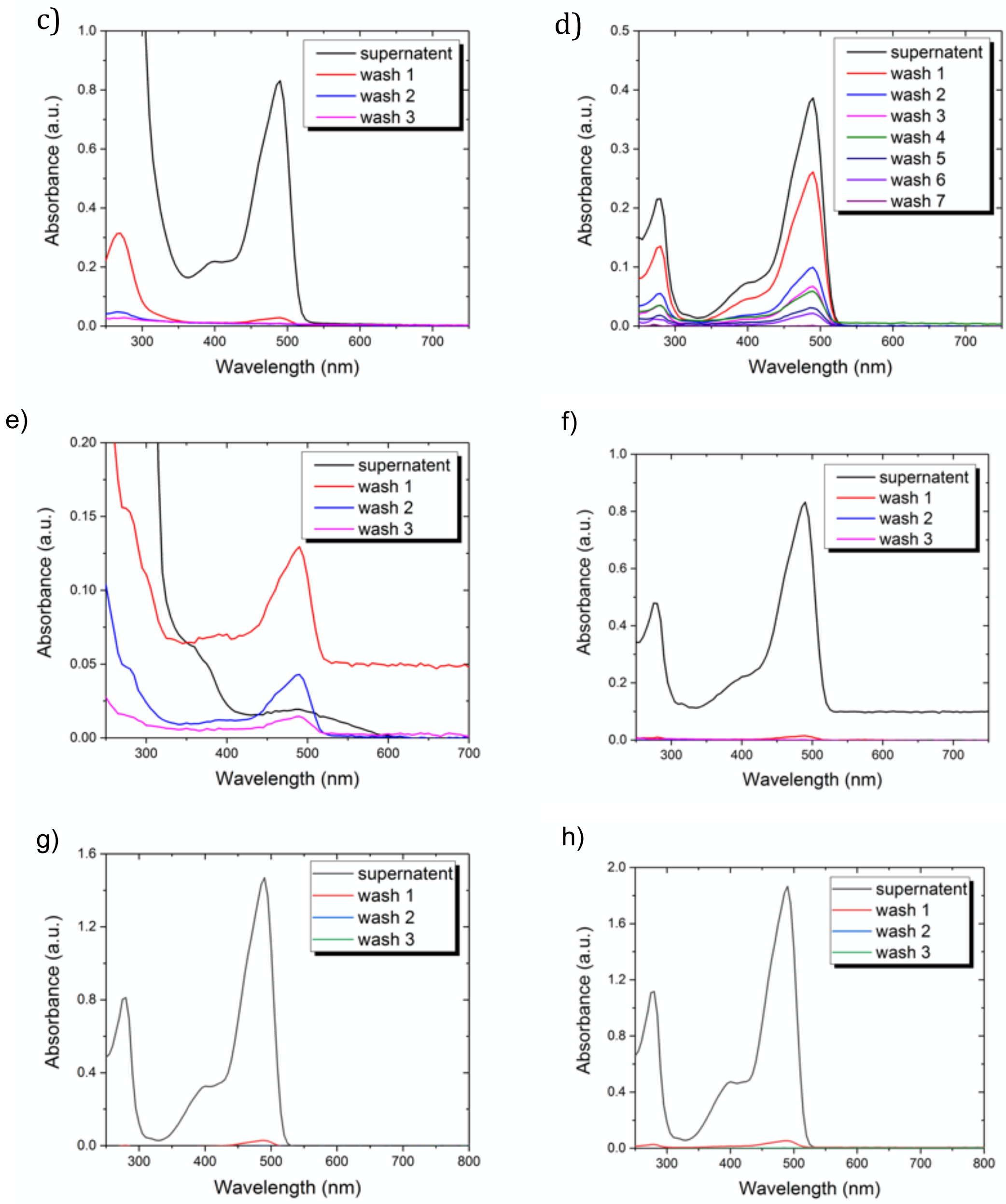

Figure S14: The UV-vis spectra of the products from the GFP attachment reactions, all spectra recorded in PBS (100 mM, pH 7.4) at a concentration of 1 mg/mL. a) AHAMHAOAYbTm_GFP, b) APTES475_GFP, c) PEIYbTm_GFP, d) DMSAYbTm_GFP, e) MHAYbTm_GFP, f) CysteineYbTm_GFP, f) MPS475_GFP, g) MPTMS475_GFP. It should be noted that the feature at 380 nm in the spectra for MHAYbTm_GFP, MPS475_GFP and MPTMS475_GFP is due to a lamp change in the machine used to collect the data and is not due to the UCP or GFP.

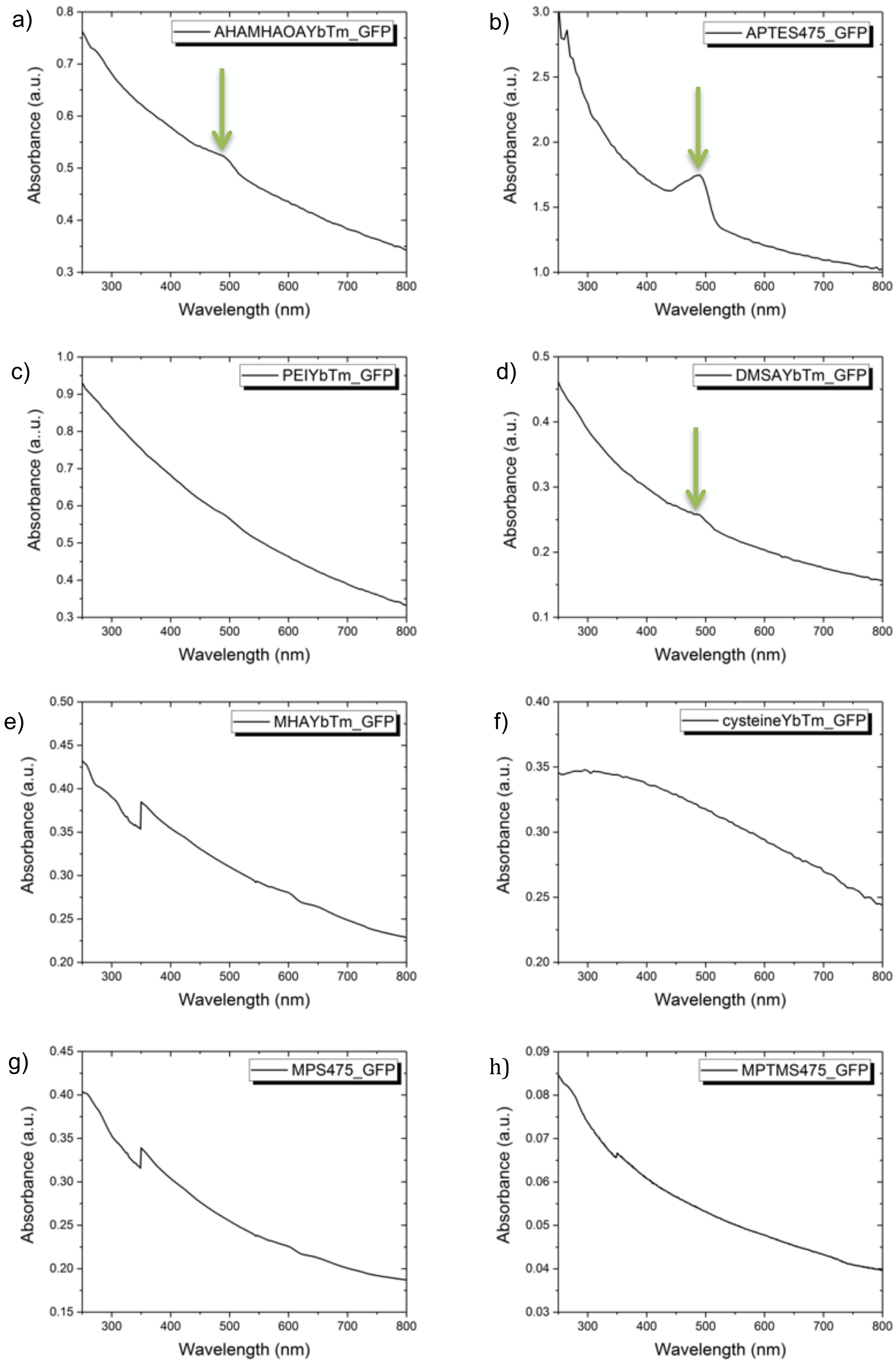
a)
AHAMHAOAYbTm_GFP
Absorbance (a.u.)
Wavelength (nm)
b)
APTES475_GFP
Absorbance (a.u.)
Wavelength (nm)
c)
PEIYbTm_GFP
Absorbance (a..u.)
Wavelength (nm)
d)
DMSAYbTm_GFP
Absorbance (a.u.)
Wavelength (nm)
e)
MHAYbTm_GFP
Absorbance (a.u.)
Wavelength (nm)
f)
cysteineYbTm_GFP
Absorbance (a.u.)
Wavelength (nm)
g)
MPS475_GFP
Absorbance (a.u.)
Wavelength (nm)
h)
MPTMS475_GFP
Absorbance (a.u.)
Wavelength (nm)

Figure S15: Solid-state reflectance spectra of the products from GFP attachment reactions. Samples were drop cast and dried between two glass slides before recording spectra. These figures show GFP (black line) the UCP (red line), and the resulting UCP_GFP conjugate (blue line). The UCPs in question are a) AHAMHAOAYbTm, b) APTES475, c) PEIYbTm, d) DMSAYbTm, e) MHAYbTm, f) CysteineYbTm, g) MPS475 and h) MPTMS475

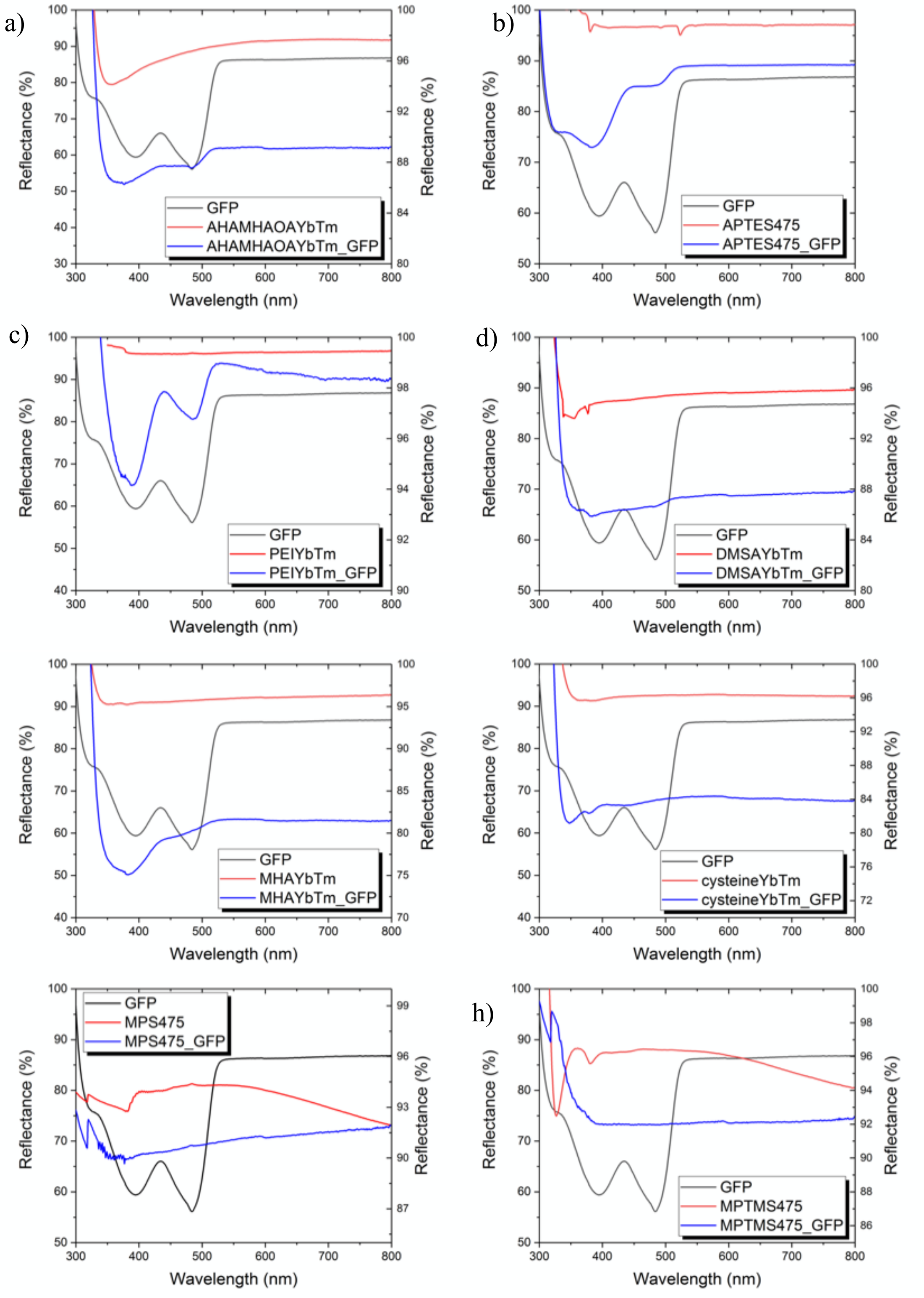

Figure S16: The 475 nm emission band for all covalent attachment reactions before (black line) and after (red line) the attachment of GFP. All spectra recorded in a 1 cm cuvette in PBS (100 mM, pH 7.4) at a concentration of 1 mg/mL, with excitation at 980 nm. All spectra have been normalized to the 800 nm band (not shown). The UCPs in question are a) AHAMHAOAYbTm, b) APTES475, c) PEIYbTm, d) DMSAYbTm, e) MHAYbTm, f) CysteineYbTm, g) MPS475 and h) MPTMS475

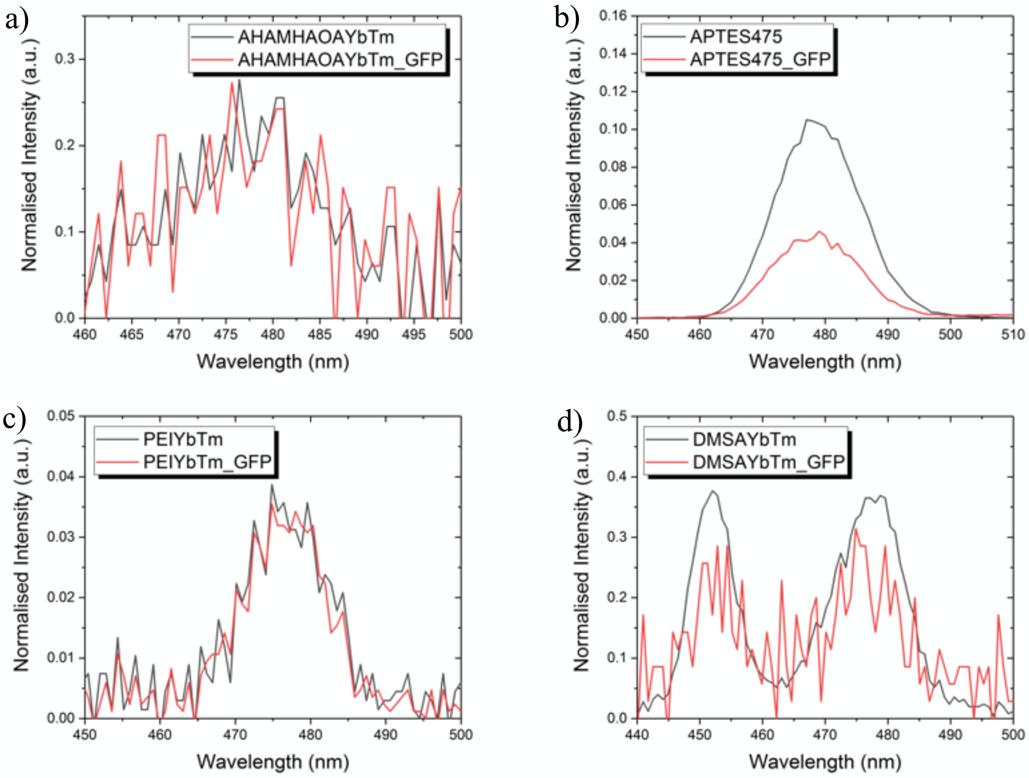

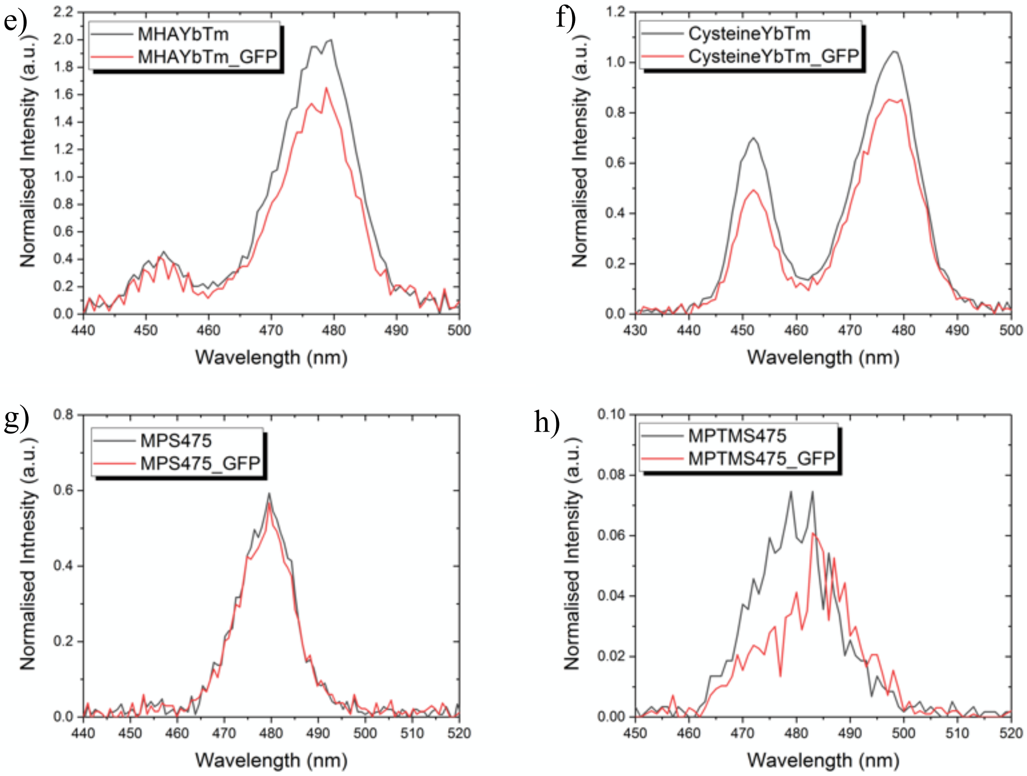

Figure S17: Comparison of the FTIR spectrum of GFP (red line), with the products from the GFP attachment reactions (black line). The UCPs in question are a) AHAMHAOAYbTm, b) APTES475, c) PEIYbTm, d) DMSAYbTm, e) MHAYbTm, f) CysteineYbTm, g) MPS475 and h) MPTMS475

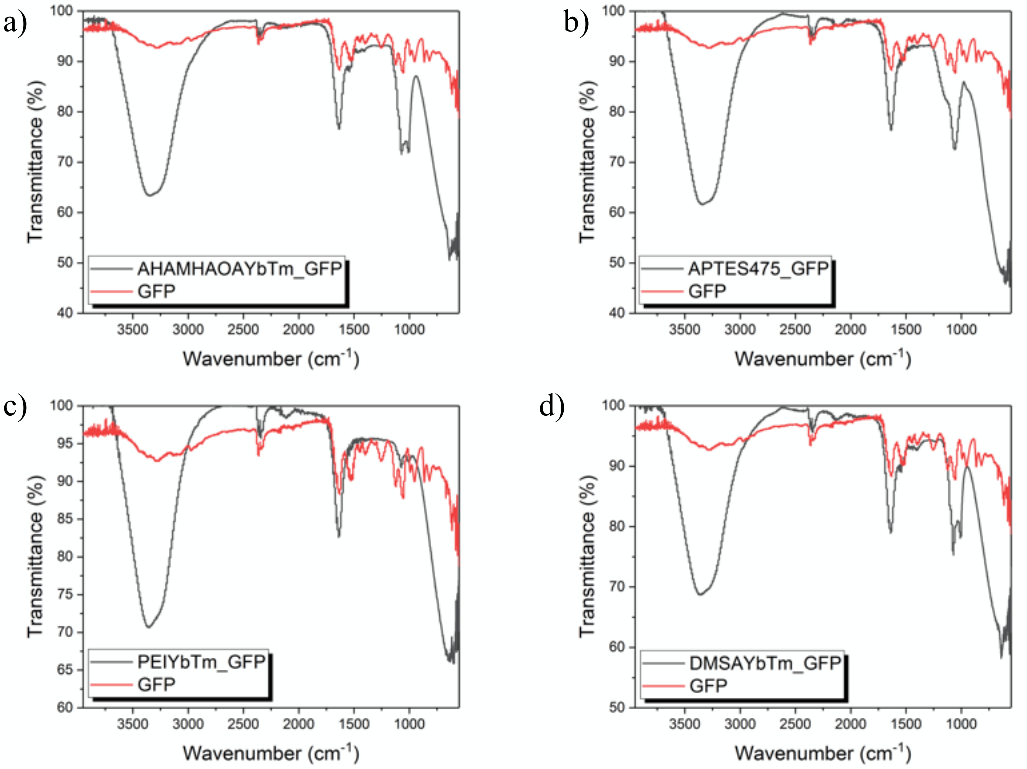

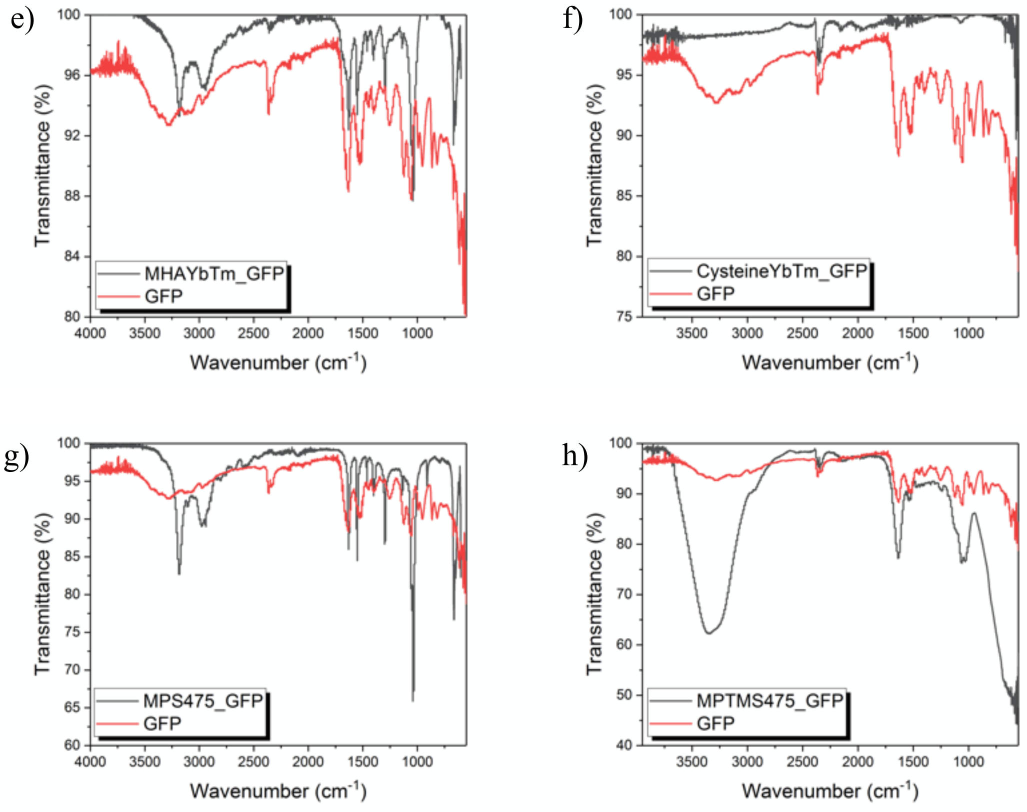

Figure S18: Comparison of the Raman spectrum of GFP (black line) with the precursor UCPs (red line) and the products from the GFP attachment reactions (blue line). The UCPs in question are a) AHAMHAOAYbTm, b) APTES475, c) PEIYbTm, d) DMSAYbTm, e) MHAYbTm, f) CysteineYbTm, g) MPS475 and h) MPTMS475

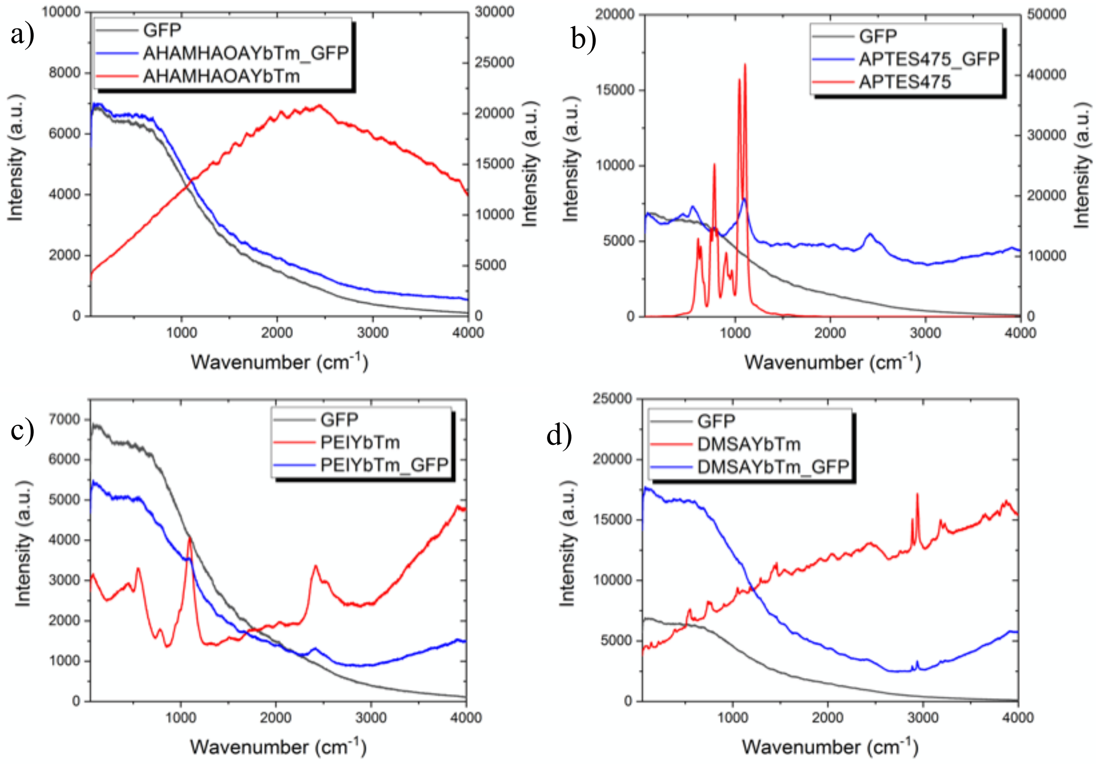

e)

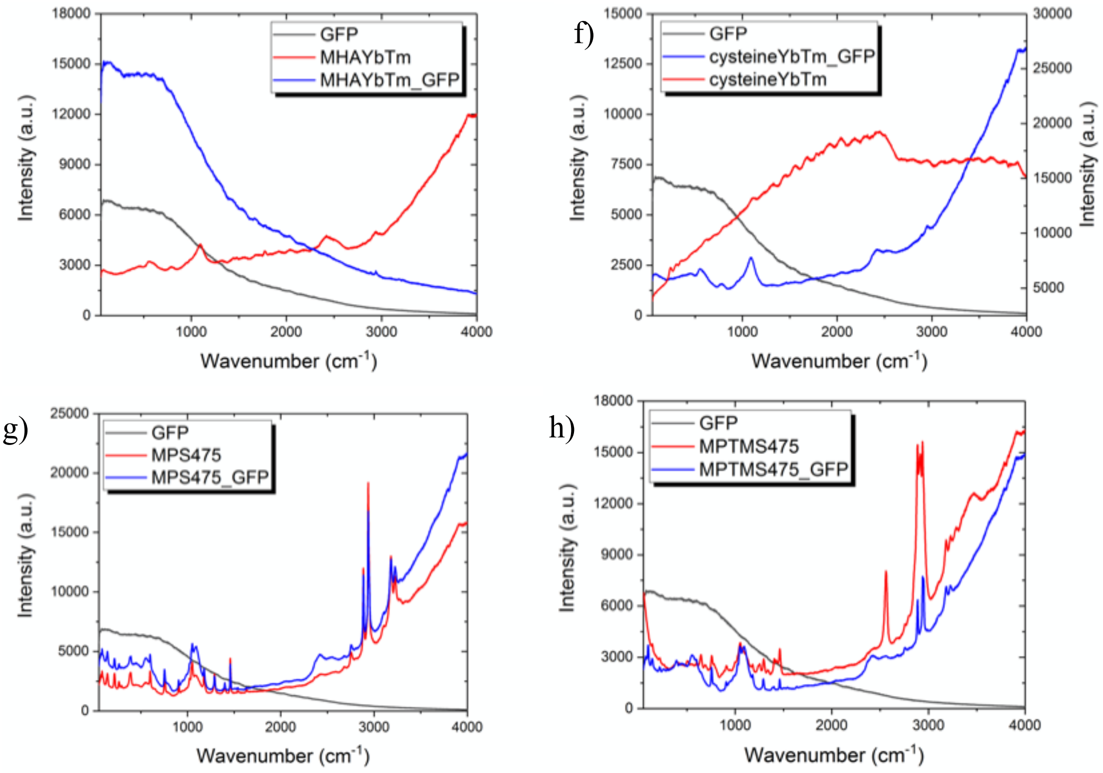

Figure S19: Thermogravimetric analysis of precursor UCPs (black line) and the products from the GFP attachment (red line). The UCPs in question are a) AHAMHAOAYbTm b) PEIYbTm c) DMSAYbTm d) cysteineYbTm e) MHAYbTm. Results summarized in the table below.

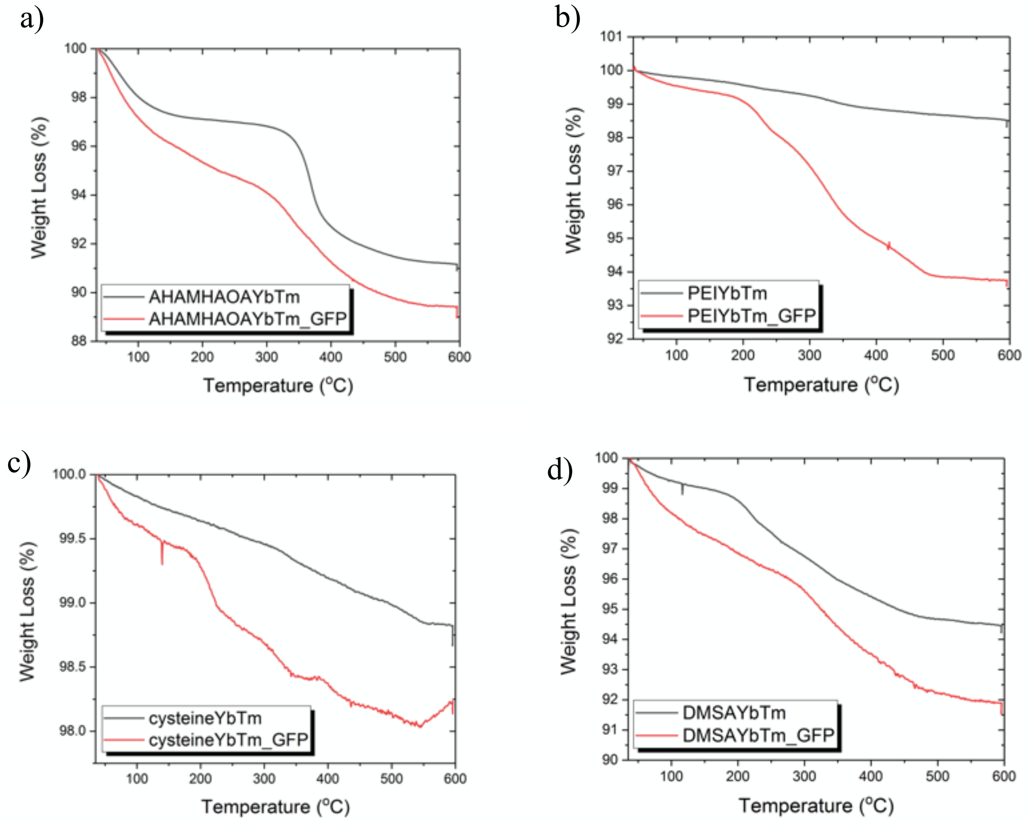

e)

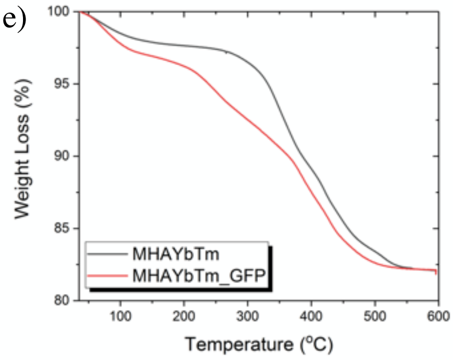

	STEP 1		STEP 2		STEP 3		STEP 4	
	T (°C)	Weight Loss (%)	T (°C)	Weight Loss (%)	T (°C)	Weight Loss (%)	T (°C)	Weight Loss (%)
AHAMHAOAYbTm	81.9	2.94	371	5.81	596	0.310		
AHAMHAOAYbTm_GFP	90.6	5.40	367	5.14	595	0.537		
PEIYbTm	156	0.575	336	0.585	572	0.443		
PEIYbTm_GFP	66.8	0.754	225	1.40	323	2.71	446	1.51
DMSAYbTm	75.9	0.867	220	1.78	371	2.79		
DMSAYbTm_GFP	98.6	3.45	365	4.76				
CysteineYbTm	321	1.17	589	0.155				
CysteineYbTm_GFP	76.7	0.152	214	0.649	341	0.612		
MHAYbTm	82.8	2.27	348	7.98	433	5.09	513	2.05
MHAYbTm_GFP	81.2	3.11	261	5.80	415	8.79		

Figure S20: Summary of DLS data for the UCP-GFP conjugates

UCP system	z-average (nm)	PDI	Zeta-potential (mv)
AHAMHAOAYbTm	12617	1.00	-10.3
AHAMHAOAYbTm_GFP	3101	0.981	-6.50
APTES475	4964	0.767	-11.1
APTES475_GFP	1183	0.395	-3.95
PEIYbTm	2730	0.583	-8.81
PEIYbTm_GFP	2037	0.677	-5.90
DMSAYbTm	4906	0.518	-6.22
DMSAYbTm_GFP	5413	0.795	-1.00
MHAYbTm	3964	0.879	-27.2
MHAYbTm_GFP	7138	0.401	-9.98
cysteineYbTm	9266	0.617	-9.03
cysteineYbTm_GFP	27390	0.704	-9.80
MPS475	5071	0.572	-10.7
MPS475_GFP	5346	0.801	-14.1
MPTMS475	5284	0.363	-14.7
MPTMS475_GFP	1840	0.213	-4.51

Figure S21: TEM images of the UCPs before (left) and after (right) the GFP attachment reactions. a) AHAMHAOAYbTm b) APTES475, c) PEIYbTm, d) DMSAYbTm, e) MHAYbTm, f) CysteineYbTm, g) MPS475 and h) MPTMS475. Summary of size data in the table below.

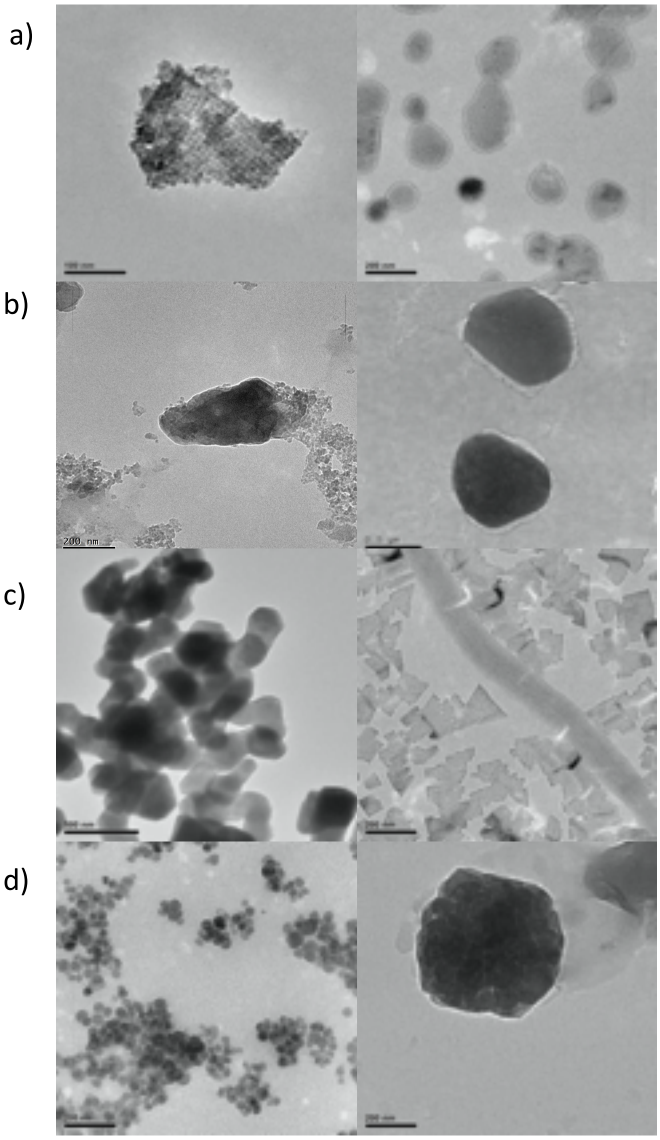
a)
b)
c)
d)

e)
f)
g)
h)

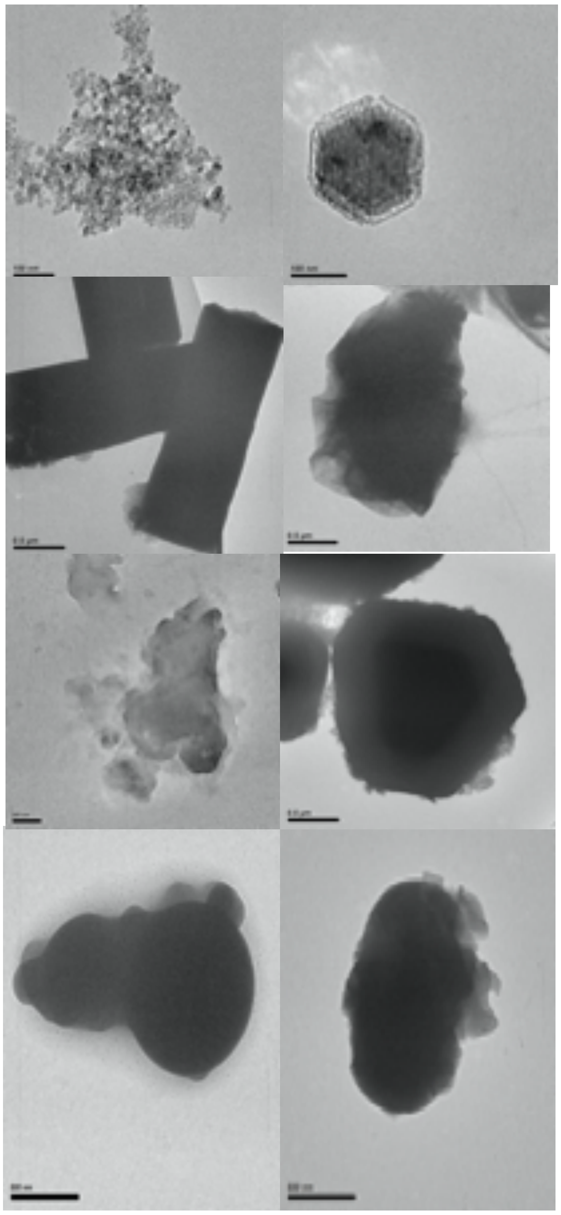

**UCP system**	**Average size calculated from TEM (nm)**
AHAMHAOAYbTm	10.7 x 10.6
AHAMHAOAYbTm_GFP	162 x 184
APTES475	810 x 680
APTES475_GFP	868 x 752
PEIYbTm	284 x 288
PEIYbTm_GFP	88 x 67
DMSAYbTm	41 x 42

DMSAYbTm_GFP	644 x 636
MHAYbTm	21 x 22
MHAYbTm_GFP	367 x 360
cysteineYbTm	2580 x 980
cysteineYbTm_GFP	1591 x 1512
MPS475	711 x 1185
MPS475_GFP	1173 x 1150
MPTMS475	543 x 554
MPTMS475_GFP	944 x 969

Figure S22: a) UV-vis of the supernatants from the covalent attachment of BM3Heme to APTES545 (10 mM PBS, pH 7.4), b) UV-Vis spectra of the washes from the cytochrome C attachment to APTES545 (100 mM PBS, pH 7.4), c) UV-Vis of the supernatants from the covalent attachment of GO to APTES475 (100 mM PBS, pH 7.4), d) UV-Vis of the supernatants from the covalent attachment of GR to APTES475 (10 mM PBS, pH 7.4)

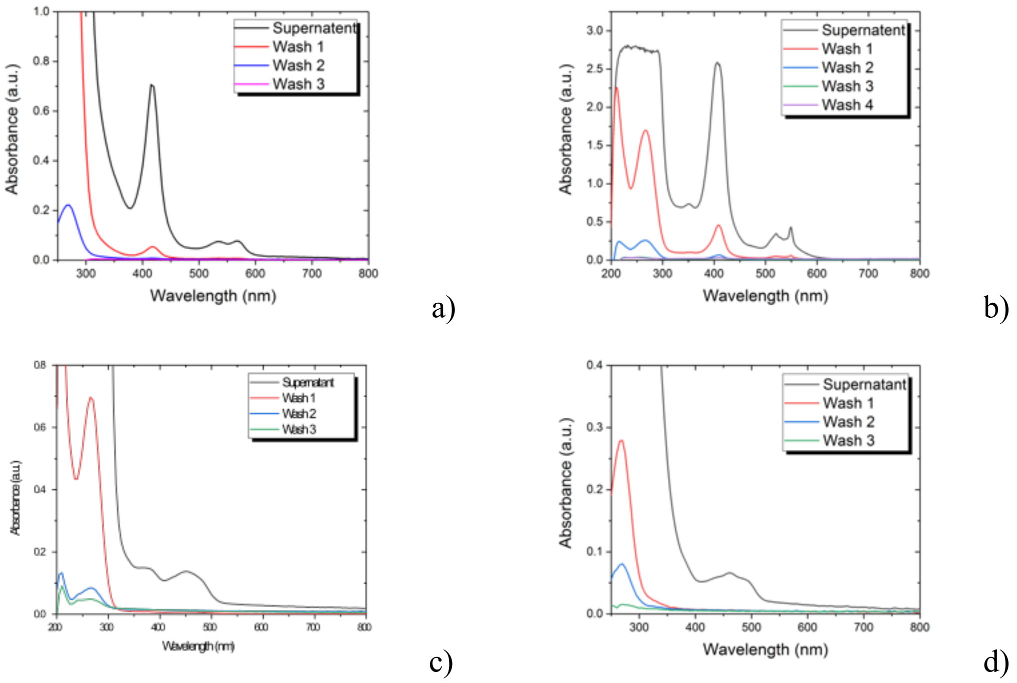

Figure S23: a) TEM image ofBM3Heme545, b) TEM image of cytC545, c) TEM of GO475, d) TEM image of GR475. Average sizes are summarized in the table below.

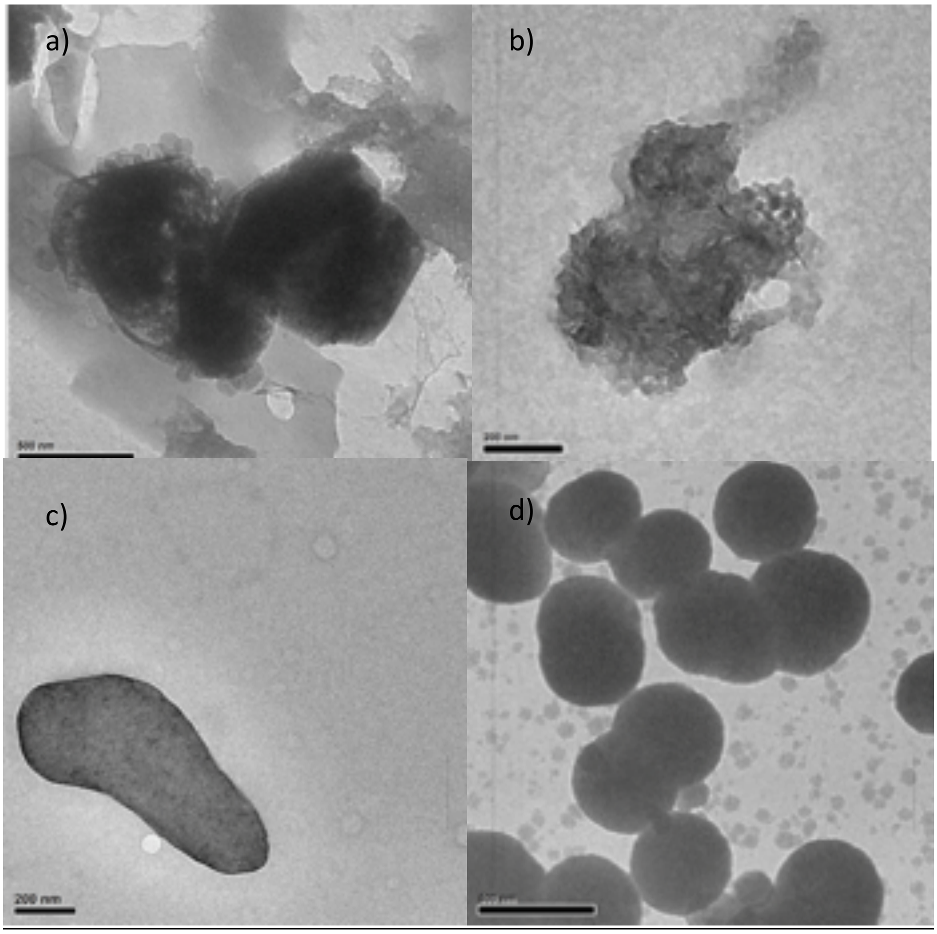

UCP system	Size calculated from TEM (nm)
PTIR-475	765
PTIR-545	320
APTES475	809
APTES545	1726
BM3Heme545	650
cytC545	600
GO475	680
GR475	520

Figure S24: a) The UV-vis spectrum of oxidised and reduced cytochrome C (0.01 M) in PBS (100 mM, pH 7.4).

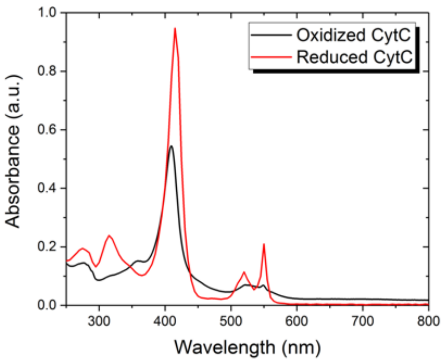

Figure S25: The normalised emission spectra of: a) APTES545 and BM3Heme545 showing only the 545 nm band, b) APTES545 and cytC545 showing only the 545 nm band, c) APTES475 and GO475 showing only the 475 nm band, d) APTES475 and GR475 showing only the 475 nm band. All samples were 1 mg/mL in in PBS buffer (100 mM, pH 7.4). All spectra have been recorded following excitation at 980 nm and are reported without correcting for the detector response. Figures a and b have been normalised to the 660 nm band, and figures c and d have been normalised to the 800 nm band.

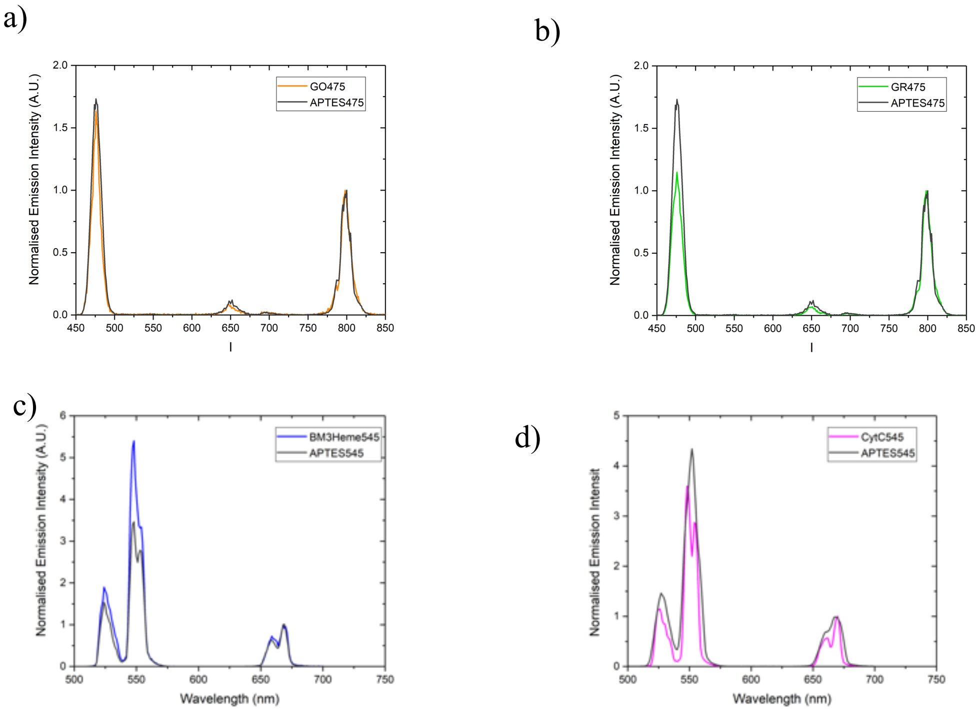

Figure S26: Summary of the DLS data for the UCP-biomolecule conjugates

UCP system	Z average (nm)	PDI	ZP
PTIR-475	1432	0.358	2.67
PTIR-545	2964	0.823	19.3
APTES475	4964	0.767	-11.1
APTES545	610	0.396	-2.63
BM3Heme545	2355	0.310	-38.8
cytC545	6850	1.00	-27.5
GO475	8785	0.657	-5.13
GR475	3484	0.657	-16.4

Figure S27: a) The solid-state reflectance spectra of BM3Heme and BM3Heme545, b) The solid-state reflectance spectra of cytC and cytC545, c) The solid-state reflectance spectra of GO and GO475, and d) The solid-state reflectance spectra of GR and GR475

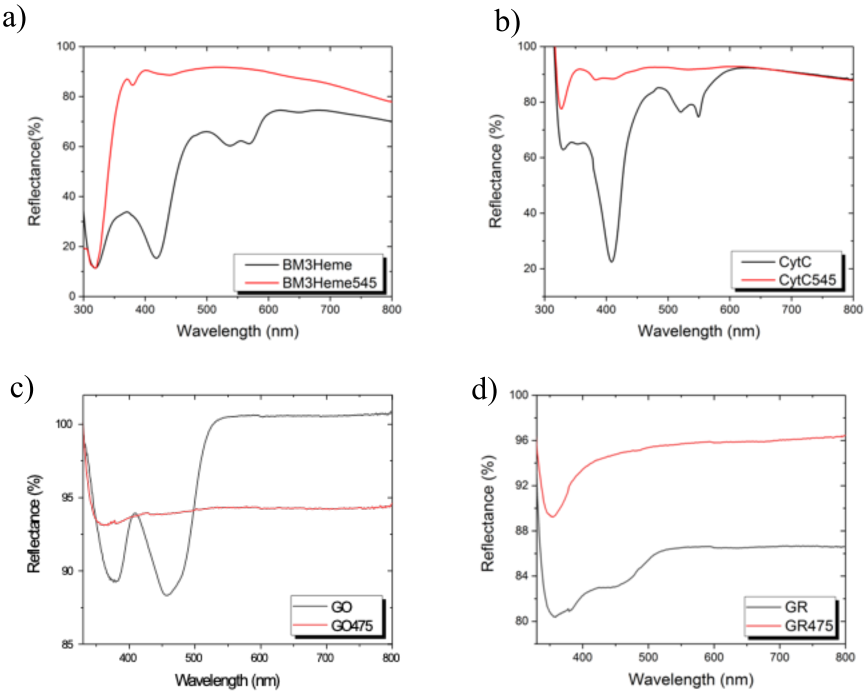

Figure S28: a) The Raman spectra of APTES5455, BM3Heme545 and BM3Heme, b) The Raman spectra of APTES545, cytC545 and cytC, c) The Raman spectra of APTES475, GO475 and GO, and d) The Raman spectra of APTES475, GR475 and GR.

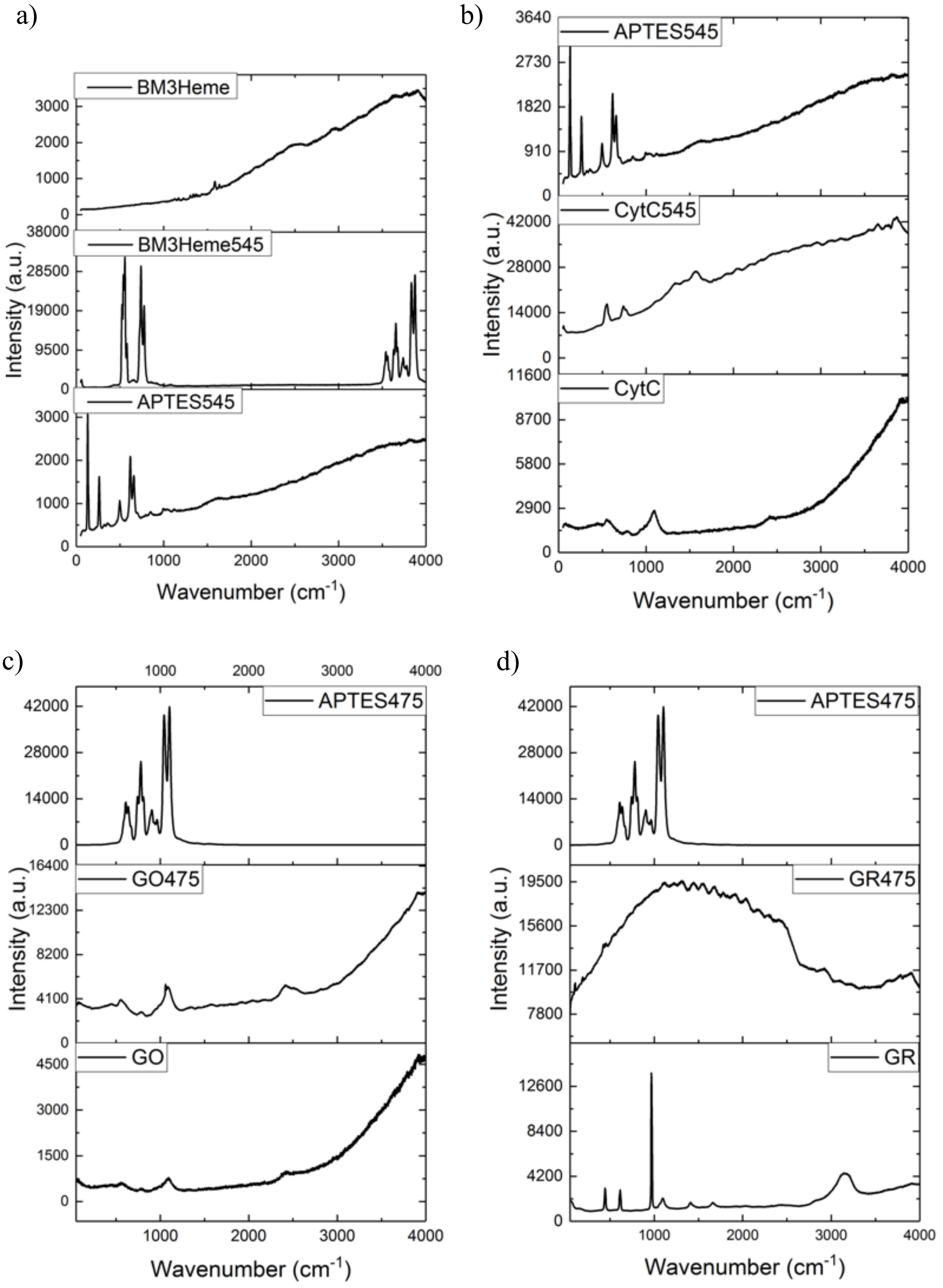

Figure S29: a) The emission spectra of: a) silica475 and MPTMS475 (1 mg/mL in 100 mM TRIS pH 7) CW excitation at 980 nm. Both spectra have been normalised to the 800 nm band. b) silica545 and MPTMS545 (1 mg/mL in 100 mM TRIS pH 7) CW excitation at 980 nm. Both spectra have been normalised to the 660 nm band. All spectra are reported uncorrected with respect to the detector response.

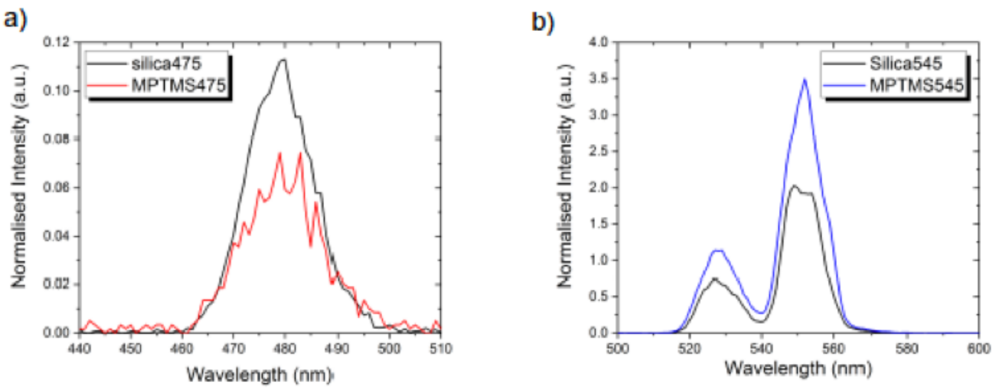

Figures regarding characterisation of PTIR-475 and PTIR-545 UCPs and functionalized UCP systems prior to bioconjugation

Figure S30: Powder X-ray Diffraction spectrum of PTIR545, PTIR475 and hexagonal phase β-Gd2SO4 (ICSD No. 636114).

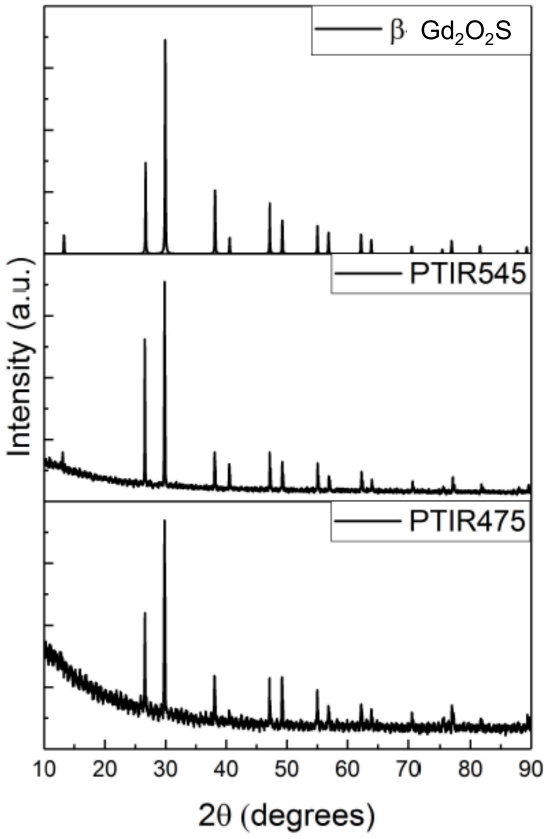

Figure S31: a) IR spectrum of MPS and MPS475, and b) Raman spectrum of MPS and MPS475.

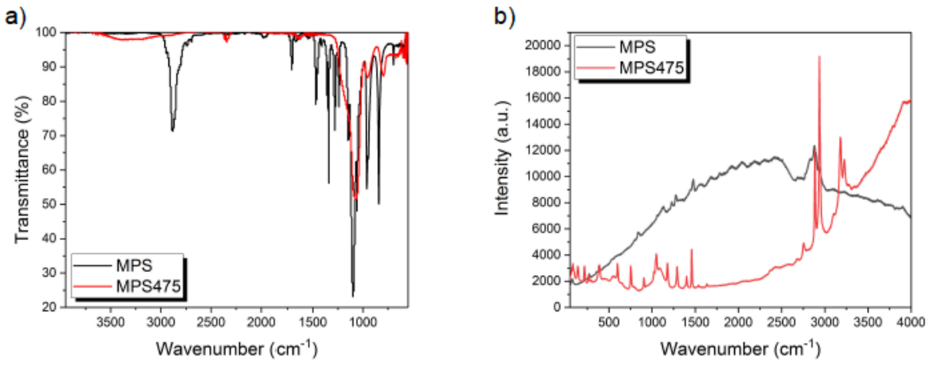

Figure S32: a) The IR spectra of 6-aminohexanoic acid, 6-maleimidohexanoic acid, oleic acid and AHAMHAOAYbTm, b) The Raman spectra of 6-aminohexanoic acid, 6-maleimidohexanoic acid, oleic acid and AHAMHAOAYbTm.

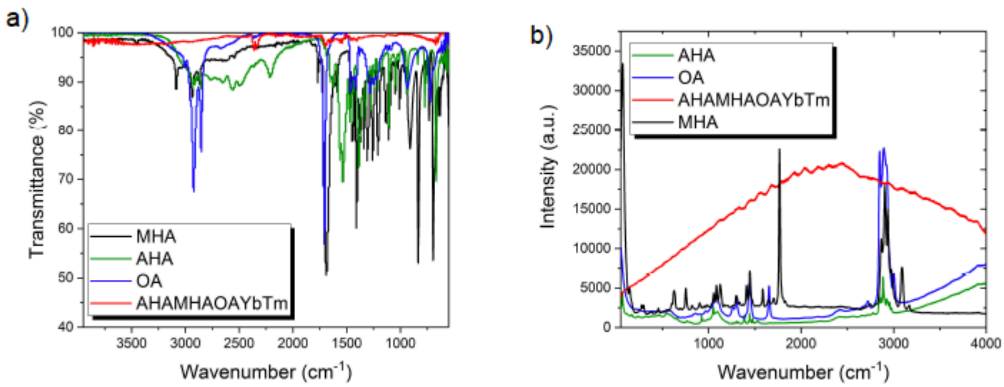

Figure S33: a) The IR spectra of 6-maleimidohexanoic acid and MHAYbTm, b) The Raman spectra of 6-maleimidohexanoic acid and MHAYbTm.

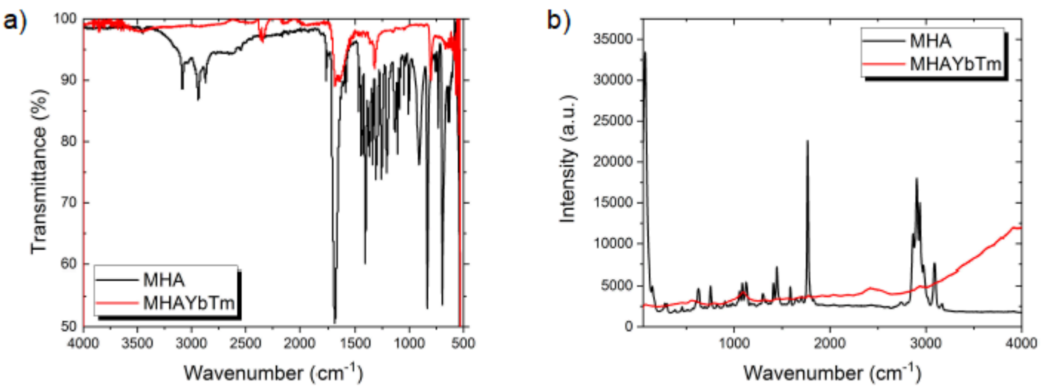

Figure S34: a) The IR spectra of polyethylenimine acid and PEIYbTm, b) The Raman spectra of polyethylenimine acid and PEIYbTm.

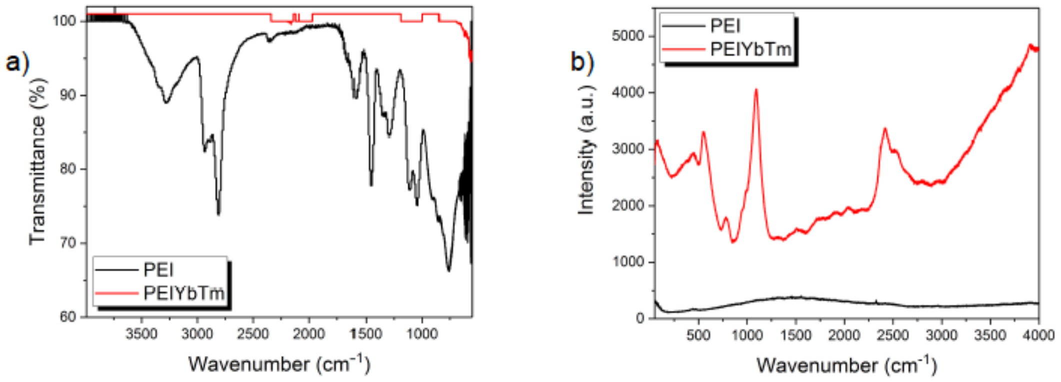

Figure S35: a) The IR spectra of DMSA and DMSAYbTm, b) The Raman spectra of DMSA and DMSAYbTm.

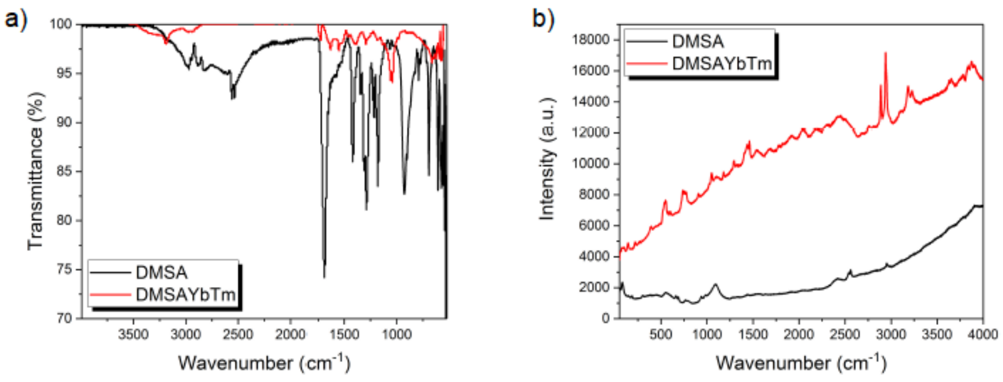

Figure S36: a) The IR spectra of cysteine and cysteineYbTm, b) The Raman spectra of cysteine and cysteineYbTm.

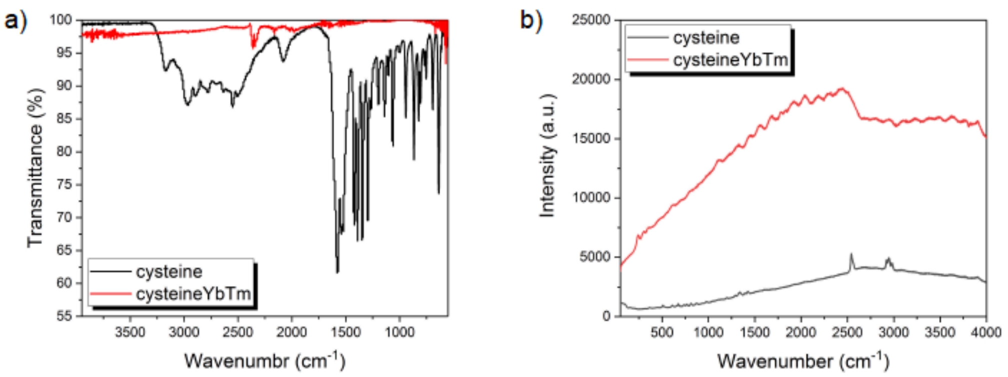

Figure S37: a) The IR spectra of MPTMS, MPTMS475 and MPTMS545, and b) The Raman spectra of MPTMS, MPTMS475 and MPTMS545.

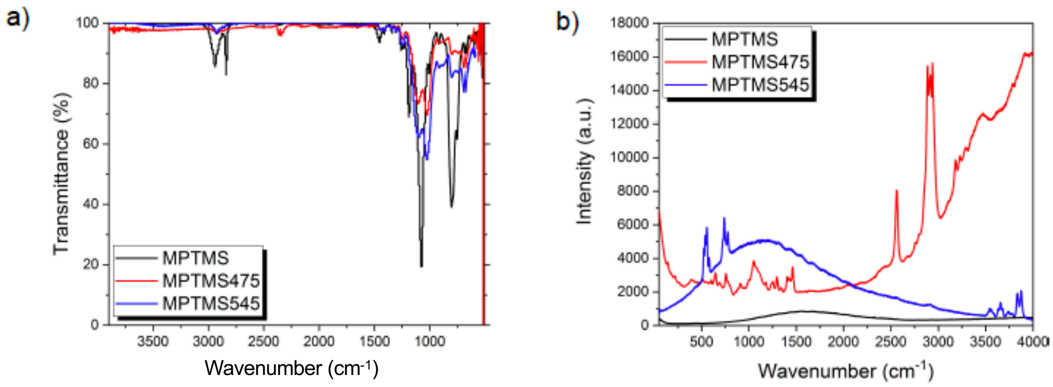

Figure S38: a) The IR spectra of APTES475 and APTES545, and b) The Raman spectra of APTES475 and APTES545.

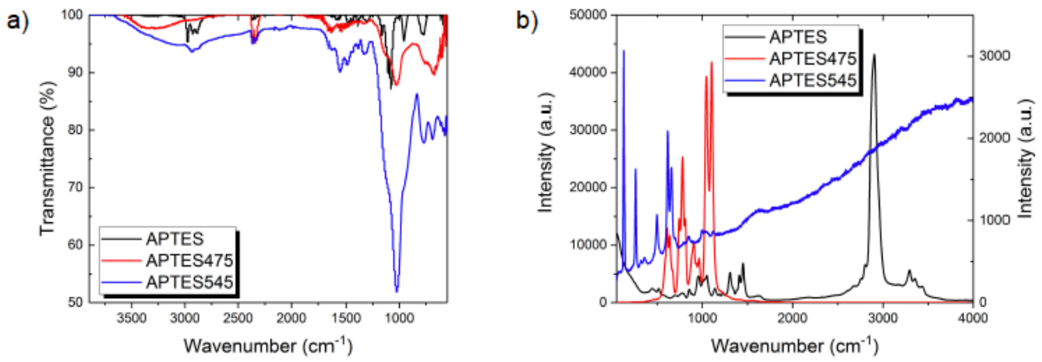

Supplement: Supplementary file 1 [file Data_Sheet_1.PDF]
